# High-content ductile coherent nanoprecipitates achieve ultrastrong high-entropy alloys

**DOI:** 10.1038/s41467-018-06600-8

**Published:** 2018-10-03

**Authors:** Yao-Jian Liang, Linjing Wang, Yuren Wen, Baoyuan Cheng, Qinli Wu, Tangqing Cao, Qian Xiao, Yunfei Xue, Gang Sha, Yandong Wang, Yang Ren, Xiaoyan Li, Lu Wang, Fuchi Wang, Hongnian Cai

**Affiliations:** 1 School of Materials Science and Engineering, Beijing Institute of Technology, 100081 Beijing, China; 2 Beijing National Laboratory for Condensed Matter Physics, Institute of Physics, Chinese Academy of Sciences, 100190 Beijing, China; 3 Herbert Gleiter Institute of Nanoscience, Nanjing University of Science and Technology, 210094 Nanjing, China; 4 State Key Laboratory for Advanced Metals and Materials, University of Science and Technology Beijing, 100083 Beijing, China; 5 X-ray Science Division, Argonne National Laboratory, Argonne, IL 60439 USA; 6 Center for Advanced Mechanics and Materials, Applied Mechanics Laboratory, Department of Engineering Mechanics, Tsinghua University, 100084 Beijing, China

## Abstract

Precipitation-hardening high-entropy alloys (PH-HEAs) with good strength−ductility balances are a promising candidate for advanced structural applications. However, current HEAs emphasize near-equiatomic initial compositions, which limit the increase of intermetallic precipitates that are closely related to the alloy strength. Here we present a strategy to design ultrastrong HEAs with high-content nanoprecipitates by phase separation, which can generate a near-equiatomic matrix in situ while forming strengthening phases, producing a PH-HEA regardless of the initial atomic ratio. Accordingly, we develop a non-equiatomic alloy that utilizes spinodal decomposition to create a low-misfit coherent nanostructure combining a near-equiatomic disordered face-centered-cubic (FCC) matrix with high-content ductile Ni_3_Al-type ordered nanoprecipitates. We find that this spinodal order–disorder nanostructure contributes to a strength increase of ~1.5 GPa (>560%) relative to the HEA without precipitation, achieving one of the highest tensile strength (1.9 GPa) among all bulk HEAs reported previously while retaining good ductility (>9%).

## Abstract (web summary)

High entropy alloys usually emphasize equiatomic compositions, which restrict the compositions available to induce strengthening via precipitation. Here the authors use spinodal decomposition in a five-element alloy to obtain high content nanophases and the highest tensile strength reported to date.

## Introduction

**Fig. 1:**
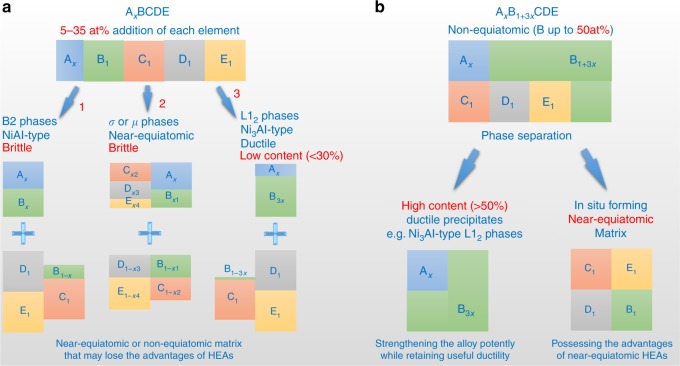
Comparison between the near-equiatomic alloying concept of HEAs and our strategy. **a** The near-equiatomic concept hinders the development of stronger and ductile HEAs because (i) near-equiatomic ratios prefer to form brittle phases (generally near-equiatomic, e.g. paths 1 and 2), and (ii) even though decreasing the Al concentrations can increase Ni/Al ratios and obtain ductile L1_2_ phases (path 3), near-equiatomic compositions limit the Ni concentrations and hence the L1_2_ phase contents. **b** Our alloying strategy aims to obtain a final microstructure combining a near-equiatomic matrix with high-content ductile precipitates, regardless of the initial atomic ratio

High-entropy alloys (HEAs), which contain at least four principal elements in (near-)equiatomic ratios, have attracted extensive attention because of their interesting properties^1–6^. In the early stage of the development of HEAs, researchers wanted to seek single-phase solid-solution alloys because they considered that intermetallics are brittle and may degenerate the properties of HEAs^1–4^. However, the fact that in most engineering alloys, secondary phases contribute significantly to the alloy properties is also true in HEAs; many reported HEAs that can overcome the strength–ductility trade-off contain two or more phases^7–17^. Proper control of the type, shape, size, volume fraction, and distribution of precipitation phases is critical for the development of the HEAs with high strength while retaining sufficient ductility^6–8,13–17^. Ni_3_Al-type ordered L1_2_ phases, which are ductile and coherent with FCC matrices^18^, are one important class of strengthening phases in FCC-based alloys such as superalloys, and the strengthening effects depend prominently on the volume fractions of these precipitates^19–21^. Similar to conventional FCC alloys, the HEAs strengthened by L1_2_ precipitates also possess good balances between strength and ductility^5,13–17^. However, the current alloying concept of HEAs, which emphasizes near-equiatomic initial compositions, intrinsically limits the increase in the contents of these ductile intermetallic strengthening phases^7,8,13–17^. As schematically shown in Fig. 1a, near-equiatomic HEAs tend to form brittle intermetallics (generally near-equiatomic, e.g. NiAl-type B2 and complex σ phases)^7,8,13^; formation of ductile L1_2_ phases requires a high Ni/Al ratio. As a result, inclusion of L1_2_ precipitates into FCC HEAs has to decrease the concentration of the L1_2_ forming elements to a value <7 at% in order to obtain the near-equiatomic matrices, e.g. Al_0.3_CoCrFeNi^13^ and (CoCrFeNi)_94_Ti_2_Al_4_^17^. In other words, based on the alloying strategy/concept of HEAs, a high Ni/Al ratio that promotes the formation of L1_2_ phases can only be achieved by reducing the L1_2_ former because the Ni additions in near-equiatomic HEAs are commonly <25 at%. Such low concentrations of the L1_2_ former lead to difficulty in developing stronger HEAs with large L1_2_ volume fractions^14–17^.

To obtain a HEA with high-content L1_2_ strengthening phases, we use a different way to increase the Ni/Al ratio by adding more Ni element rather than reducing Al concentration. This composition design aims to ensure that the phase separation can generate a near-equiatomic FCC matrix in situ while forming high-content L1_2_ phases, creating PH-HEAs with superior strength (Fig. 1b). Based on this strategy, we develop a non-equiatomic system Al_0.5_Cr_0.9_FeNi_2.5_V_0.2_, which has a high Ni concentration of ~50 at% and a high Ni/Al ratio of ~5. Moreover, our alloy excludes Co to reduce the cost and contains ~4 at% V to stabilize and strengthen L1_2_ phases^20^. This well-designed non-equiatomic system can form a PH-HEA in situ by utilizing spinodal decomposition to create a low-misfit coherent nanostructure composed of a near-equiatomic disordered FCC matrix and high-content ordered L1_2_ nanoprecipitates (>50 vol%). This distinctive spinodal order–disorder nanostructure can resist dislocation motion by providing strong diffuse obstacles and by creating antiphase boundaries (APBs) on the slip planes of these ordered L1_2_ nanophases^19^, contributing to an ultrahigh strength while retaining good ductility and work-hardening capacity. We anticipate that this alloying strategy can offer more possibilities for the compositional design of HEAs, and will open new avenues for development of ultrastrong and ductile HEAs superior to existing commercial alloys.

## Results

### Material properties

**Fig. 2:**
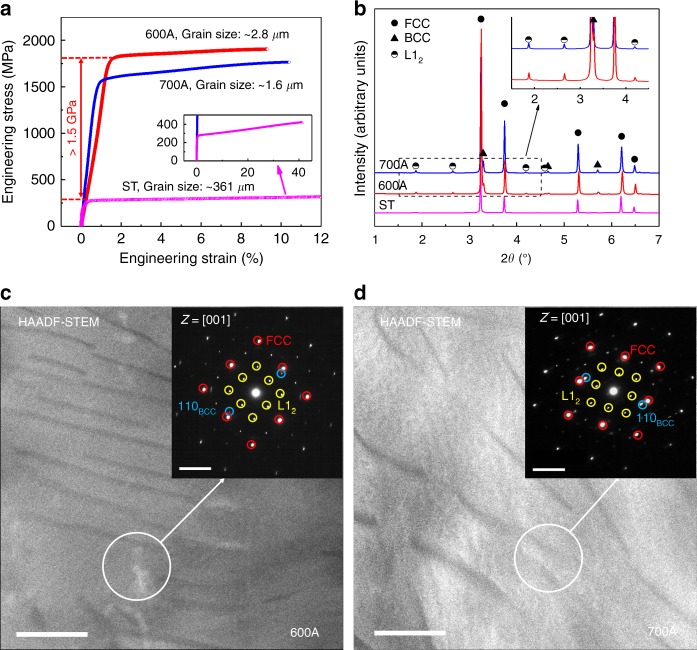
Mechanical properties and phase constituent of our alloy prepared under various processing conditions. **a** Tensile engineering stress–strain curves showing remarkable increases in strength occurring after cold-rolling and ageing (>1500 MPa for 600 A and ~1200 MPa for 700 A compared with the ST sample). **b** HEXRD spectra showing that the aged samples contain FCC, L1_2_, and BCC phases, whereas the ST sample is a single-phase FCC alloy. **c**, **d** HAADF-STEM images of 600 A and 700 A, respectively. Scale bar, 200 nm. The SAED patterns in the insets verify the phase composition in the aged samples. Scale bar, 5 nm^−1^

Tensile engineering stress–strain curves (Fig. 2a) show that although the solution-treated (ST) sample is soft (yield strength of ~274 MPa), remarkable strength increases occur in the samples after 72% cold rolling followed by ageing for 1 h at 600 °C and 700 °C (hereinafter named 600 A and 700 A, respectively). The maximum strengthening observed in 600 A is >1500 MPa (strength increment >560%), achieving ultrahigh yield strength (YS) of 1810 MPa and ultimate tensile strength (UTS) of 1905 MPa. Moreover, the YS and UTS of 700 A are 1570 MPa and 1763 MPa, respectively, which are ~1200 MPa (~430%) higher than those of the ST sample. In addition, both 600 A and 700 A exhibit good ductility (~9–10%) and excellent work-hardening capacity (uniform elongation to fracture). The exceptional combination of properties makes our alloy Al_0.5_Cr_0.9_FeNi_2.5_V_0.2_ superior to most traditional alloys for potential advanced structural applications.

### Phase constituent

After cold rolling and ageing, the grains in 600 A and 700 A become very fine (Supplementary Fig. 1). However, the strength increases resulting from grain boundaries are only 212 MPa in 600 A and 284 MPa in 700 A (see Methods and Supplementary Fig. 2 for calculated details), indicating that the ultrahigh strength of the samples arises from other mechanisms. Synchrotron high-energy X-ray diffraction (HEXRD) spectra of both 600 A and 700 A show superlattice peaks of the ordered L1_2_ structure and characteristic peaks of the body-centered-cubic (BCC) structure, in addition to characteristic peaks of an FCC-structured matrix (Fig. 2b). Quantitative analysis of the volume fraction of each phase based on the HEXRD results reveals that in the two samples, the L1_2_ phases are the major precipitates (~50% in 600 A and ~44% in 700 A), whereas the contents of the BCC phases are barely ~6%, confirming the validity of our strategy for designing PH-HEAs with high-content ductile L1_2_ strengthening phases. Interestingly, only two regions (bright matrix and dark precipitates with low volume fractions) are found in high-angle annular dark-field (HAADF) scanning transmission electron microscopy (STEM) images (Fig. 2c, d). However, selected area electron diffraction (SAED) patterns (insets in Fig. 2c, d) indicate that the samples indeed contain three phases―two symmetric spots exist near {200}_FCC_ in addition to the superlattice patterns of the FCC and L1_2_ phases. The interplanar spacings of the two spots are approximately 0.205 nm, which is consistent with the spacings of {110}_BCC_ calculated from the HEXRD spectra (~0.204 nm), suggesting that these low-content dark precipitates are of BCC structure and that the bright region consists of the FCC matrix and high-content L1_2_ strengthening phases.

### Spinodal order–disorder nanostructure

**Fig. 3:**
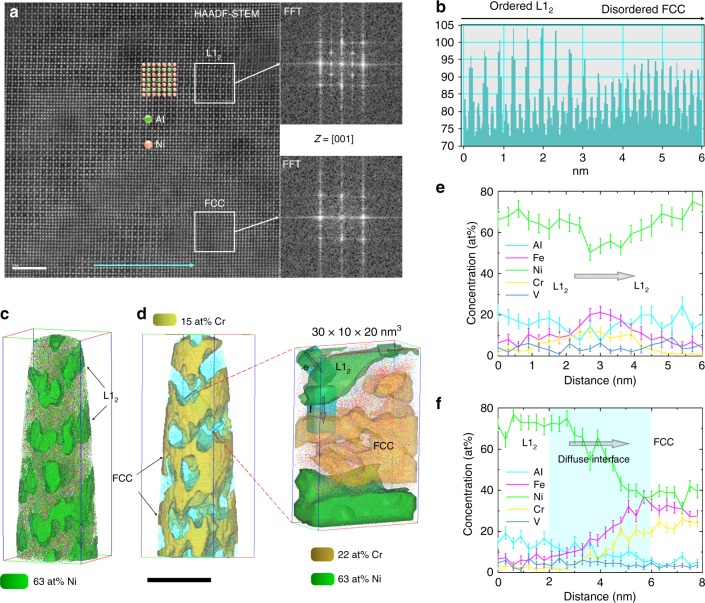
Composition, morphology and structure of phases existing in the bright matrix region of 600 A. **a** Atomic mass contrast in atomic-resolution HAADF-STEM images revealing a distinctive nanostructure consisting of a disordered FCC matrix and ordered L1_2_ phases with diffuse coherent interfaces. Scale bar, 2 nm. **b** Intensity profile along the cyan arrow marked in a showing atomic arrangement in the two phases. **c**, **d** Three-dimensional reconstruction of 63 at% Ni and 15 at% Cr isoconcentration surfaces presenting the morphologies of the ordered L1_2_ precipitates and disordered FCC matrix, respectively. A box was selected from d to provide more details about the morphology and composition of the two phases. Scale bar, 40 nm. **e**, **f** One-dimensional concentration profiles showing the element distributions from L1_2_ to L1_2_ and L1_2_ to FCC, respectively. Error bars, s.d.

To reveal the microstructure of the FCC matrix and L1_2_ phases, we conducted atomic-resolution HAADF-STEM using atomic mass contrast and atom probe tomography (APT). We find an interesting nanostructure consisting of two fully coherent nanoscale regions in atomic-resolution images (Fig. 3a), in which brighter columns represent heavier elements (e.g. Ni) whereas darker columns refer to lighter atoms (e.g. Al). The intensity profile along the cyan arrow across the two regions in Fig. 3a reveals that heavier and lighter elements are arranged alternately in one region, whereas in the other region the intensity is uniform (Fig. 3b). Fast Fourier transform (FFT) of the region with uniform intensity presents an FCC-structured diffraction pattern, whereas that of the alternately arranged region demonstrates an L1_2_-type superlattice pattern. Interestingly, the two nanophases seem to be tangled and have no abrupt changes in composition (composition continuously changing without abrupt interfaces), and the lattice parameters of the two highly coherent phases are almost the same (0.3582 nm for FCC and 0.3583 nm for L1_2_). Consequently, these L1_2_ phases are invisible in Fig. 2c, d. Hereinafter we mainly present the APT results of the detailed microstructure of 600 A because the microstructures of 600 A and 700 A are similar (Supplementary Fig. 3).

The three-dimensional morphologies of the ordered L1_2_ nanoprecipitates and disordered FCC matrix are revealed by reconstructing 63 at% Ni and 15 at% Cr isoconcentration surfaces, respectively, based on the APT data (Fig. 3c, d). The tangled nanostructure can be seen more clearly, where the interconnected disordered FCC matrix serves as the frame and is filled with the ordered L1_2_ phases (also interconnected). Interestingly, this tangled nanostructure with diffuse coherent precipitate−matrix interfaces seems to be an isotropic spinodal structure^22^. To further investigate the composition and morphology at the phase interfaces, we selected an analysis box of 30 × 20 × 10 nm^3^ from Fig. 3d and reconstructed an isoconcentration surface with a higher Cr content (22 at%) within it. The one-dimensional concentration profiles of phase interfaces (Fig. 3e, f) demonstrate that Fe slightly enriches at the L1_2_−L1_2_ interface and that all elements continuously change from FCC to L1_2_ without an abrupt FCC−L1_2_ interface (diffuse interface width ~4 nm). The one-dimensional concentration profiles across multiple FCC and L1_2_ phases also exhibit long-range periodic composition fluctuations without abrupt changes in composition (Supplementary Fig. 4). These results suggest the spinodally decomposed nature of the phase separation^22^. The mean compositions of the two phases confirm that the FCC matrix is a phase conforming to the near-equiatomic alloying concept of HEAs and that our alloying strategy for producing PH-HEAs in situ by phase separation is valid (Supplementary Fig. 4c). Moreover, the APT results indicate that the BCC phases are Cr-rich lamellar phases with the interfaces slightly enriched by Fe (Supplementary Fig. 5).

## Discussion

To form a near-equiatomic matrix in situ, we add a high content of Cr (~18 at%) into our alloy. Such a high addition promotes the change of the phase separation mechanism to spinodal decomposition^23^ and reduces the difference in the lattice parameters between the FCC matrix and L1_2_ nanophases^24^, thereby stabilizing the high-density coherent interfaces^25^ and lowering the driving force for competitive coarsening^26^. During phase separation, both Cr and Fe are rejected from the L1_2_ phases, exhibiting similar cluster trends. We observe that the Zener ratios (a dimensionless number used to quantify the anisotropy of cubic crystals; the larger the deviation from one, the stronger the anisotropy) of Cr and V are <1, whereas those of the other elements are greater than one, and Cr has the highest elastic parameters^21,27^, suggesting that such a cluster can reduce the anisotropy of the matrix and stabilize this distinctive isotropic spinodal nanostructure. Since the spinodal nanostructure is isotropic, no preferred orientation exists during phase separation, leading to no additional satellite spots appearing in the SAED patterns (Fig. 2c, d). It should be noted that phase separation phenomena including spinodal decomposition are common in HEAs^1,28–38^. It is therefore that this work can utilize them to create a PH-HEA in situ with high-content L1_2_ precipitates from a non-equiatomic system. More importantly, such a spinodal nanostructure should be closely related to the ultrahigh strength of our alloy because it contains ordered L1_2_ nanoprecipitates with high density and volume fraction, which can introduce abundant low-misfit, coherent, nanoscale interfaces^39^ that can effectively strengthen metals, e.g. nanotwins^39–41^ and nanoprecipitates^26^.

In our HEAs, the lattice constant and modulus of the ordered L1_2_ precipitates approximate to those of the disordered FCC matrix, and no new FCC−L1_2_ interfaces form after dislocations shear these L1_2_ precipitates with diffuse coherent interfaces. Therefore, strengthening by coherent strain, modulus difference, and interfacial energy increase is negligible^15–17^. Instead, the L1_2_ nanophases were formed by spinodal decomposition. This spinodal nanostructure can resist dislocation motion by providing strong diffuse attractive obstacles to trap moving dislocations and by creating APBs on the slip planes of these ordered L1_2_ nanophases when they are sheared by dislocations^19^. The contribution of the spinodal nanostructure to the YS of 600 A is calculated to be 1158 MPa (see Methods in details). Superposing additional strengthening from grain boundaries and the BCC phases, the total increase in YS is estimated to be 1524 MPa, which agrees with the experimental result of 1536 MPa, confirming that this spinodal order–disorder nanostructure can potently strengthen our alloys. Furthermore, no pile-up dislocations are found in the FCC + L1_2_ region (Supplementary Fig. 6), suggesting that the diffuse low-misfit coherent FCC−L1_2_ interfaces can minimize the elastic strain accumulation resulting from dislocation shear and hence prevent crack initiation at these interfaces, contributing to good ductility.

**Fig. 4:**
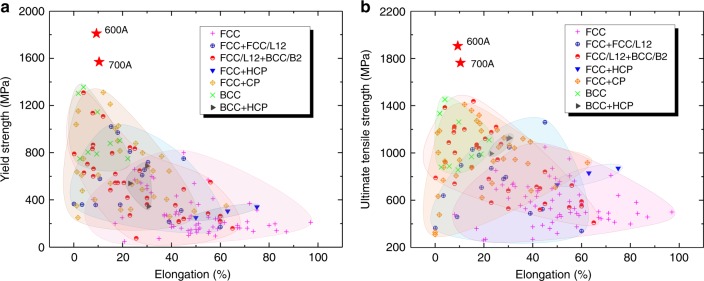
Comparison of our developed HEAs (600 A and 700 A) with existing HEAs. **a**, **b** Maps of elongation versus YS and UTS of HEAs reported previously at room temperature, respectively, where HCP and CP refer to the phases with hexagonal close-packed and complex lattice structures (e.g. σ and μ phases), respectively. The data of the tensile properties of reported HEAs were acquired from Supplementary Table 1

We compare the tensile properties of our PH-HEA with those of reported HEAs in Fig. 4. It is clear that the tensile properties depend on the phases present in alloys. FCC alloys are ductile but relatively soft, whereas BCC alloys are hard but relatively brittle, and strength–ductility combinations of multiphase alloys are generally better than those of single-phase ones. In particular, our alloys (600 A and 700 A) have significant advantages over existing bulk HEAs, including the highest YS and UTS and a good strength−ductility balance, showing the enormous potential for advanced structural applications.

Our alloying strategy not only involves the change to the initial compositions (non-equiatomic), but also requires the formation of a near-equiatomic/high-entropy solid-solution matrix in situ after separating out enough strengthening phases. The latter requirement is the primary difference relative to the previous HEA design concepts, which only consider the initial compositions regardless of whether the final primary phases are high-entropy solid solutions or not, leading to the possible loss of the HEA advantages. As a result, our alloying strategy can introduce desired strengthening phases with controllable contents while still possessing the advantages of HEAs, and the corresponding HEA has several unique advantages over previous reported HEAs, as well as other materials strengthened by abundant low-misfit coherent nanotwins^39–41^/nanoprecipitates^26^. First, our selected strengthening phases are ductile, highly coherent and low misfit with matrix (i.e. they can compatibly deform with the matrix), implying that increasing their content does not significantly degenerate the ductility. Controllable microstructure (by either alloying or processing design) provides numerous possibilities for the optimization of properties; for instance, the strength of our alloy can be controlled within a very wide range, at least, of ~270–1900 MPa, and the largest elongation is >40%. Furthermore, benefiting from the advantages of the multi-principal-element/high-entropy matrix, high Cr addition can improve the resistance to corrosion and oxidation, and high Al content reduces the alloy density. These advantages present the potential for the application of our HEA to many rigorous service conditions, such as those subjected to very large loading and involving highly corrosive environment. More importantly, the spinodal nanostructure achieves an amazing strengthening effect (>1.5 GPa), good ductility (>9%), and excellent work-hardening capacity (uniform elongation to fracture), which are higher than those induced by independent spherical hard nanoparticles reported recently (~1.1 GPa, 8.2%, and 3.8% uniform elongation, respectively)^26^ because of the higher volume fractions and the different strengthening mechanisms. It should be noted that the exciting strengthening effect of the isotropic spinodal structure with ductile Ni_3_Al-type L1_2_ nanophases in our HEA is different from that of the anisotropic spinodal structures with NiAl-type B2 phases reported previously^42,43^. The tensile properties of these reported HEAs with the anisotropic spinodal structures were unavailable and the compressive properties were just reported. The strength data obtained from the two loading modes are inappropriate to be analogized. Many HEAs that have very high compressive strength are too brittle to bear tensile loading^43–45^, leading to inferior competitiveness compared with commercial alloys. These results imply that although the spinodal structures are common in HEAs, the strengthening mechanism of anisotropic spinodal structures is different from that of the isotropic spinodal structures in our HEA. The new strengthening mechanism of the isotropic spinodal nanostructure demonstrates another classical example of metallic strengthening by low-misfit coherent nanoscale interfaces/precipitates, revealing a new strategy to potently strengthen alloys without severely losing ductility. Overall, the distinctive microstructure and excellent mechanical properties of our HEAs present the possibility of development of new HEAs with ultrahigh strength, sufficient ductility, and resistance to corrosion and oxidation to compete with traditional structural materials.

## Methods

### Samples preparation

Master Al_0.5_Cr_0.9_FeNi_2.5_V_0.2_ alloy ingots were prepared by arc-melting pure metals (Al, Cr, Fe, Ni, and V with purity >9.9%) under high-purity argon atmosphere. During preparation, the ingots were inverted and remelted at least four times to improve chemical homogeneity. These master alloy ingots were subsequently casted into a copper mold with a dimension of 50 × 13 × 30 mm^3^. To reduce elemental segregation, the as-cast alloys were solution-treated at 1200 °C for 24 h followed by water quenching. The solution-treated alloys were then cold-rolled in steps at room temperature with a total rolling reduction ratio of 72%. Finally, the as-rolled samples were aged at 600 and 700°C for 1 h followed by air cooling.

### Tensile testing

Flat, dog-bone-shaped tensile samples with a gauge length of 10 mm, a width of 3 mm and a thickness of 1 mm were cut by electrical discharge machining, and polished with 2000-grit SiC papers. Tensile tests were performed by a CMT4305 universal electronic tensile testing machine at room temperature using a nominal strain rate of 1 × 10^−3^ s^−1^.

### Microstructural characterization

Grain morphology was characterized by a Zeiss Axio Observer A1m inverted optical microscope (OM) and a JEOL JSM-7001F scanning electron microscope with a TSL OIM 6.2 system for electron backscatter diffraction (EBSD) analysis. The specimens were ground with 2000-grit SiC papers, then mechanically polished using 2.5- and 0.5-grit diamond pastes, and finally polished using oxide polish suspension. The surfaces of the OM specimens were corroded by Marble’s corrosive liquid to show grain boundaries. The EBSD scanning step is 0.05 μm, and the data are processed and analyzed by TSL OIM 6.2 software.

Phases existing in the current alloys were investigated by a synchrotron-based high-energy X-ray diffraction technique, at the 11-ID-C beam line of the Advanced Photon Source, Argonne National Laboratory, USA. The wavelength *λ* of the high energy X-ray used here is 0.011725 nm. To obtain precise lattice parameters *a*, we calculated the lattice parameters of all peaks in the HEXRD spectra, and plotted them versus *f*(*θ*) = (cos^2^*θ*/sin*θ* + cos^2^*θ*/*θ*)/2, where *θ* is the Bragg angle of the diffraction peaks^46^. We then fitted the plot using the linear least squares method and extrapolated to *f*(*θ*) = 0, i.e. *θ* = 90°, where the lattice parameter *a* is the precise value. The results showed that although the lattice parameter of the ST sample is 3.5962 ± 0.0006 Å, the lattice parameter of the FCC matrix of 600 A (3.5815 ± 0.0008 Å) with a high Cr content was decreased to a value very close to that of the L1_2_ precipitates (3.5832 ± 0.0009 Å). The ± values mean the standard deviation given by the linear least squares fitting. The mean size (particle diameter *d*) of the ordered L1_2_ precipitates was determined according to the Scherrer equation *d* = *Kλ*/*β*cos*θ*^47^, where *β* is the full width at half maximum (FWHM) of the diffraction peak (in radian) and *K* is a constant and approximates to 0.9 for cubic symmetry^48^. Many factors such as fine particle size, chemical heterogeneities, stress gradients, and microstresses can cause broadening of the (100) superlattice peak of the L1_2_ phases. Fortunately, in addition to fine particle size, other factors broaden both the superlattice and fundamental peaks. The difference between the FWHM of the (100) superlattice peak (~0.043°) and that of the fundamental peaks (~0.026°) in 600 A is the broadening resulting from the fine particle size^49^, affording a mean diameter of the L1_2_ phases of ~35 nm in 600 A. The highest peaks of FCC + L1_2_ (111) and BCC (110) are clearly overlapped. To reduce error, we performed the quantitative phase analysis based on the secondary high peak of each phase, i.e. the (200) fundamental peak of the FCC + L1_2_, the (100) superlattice peak of the L1_2_, and the (211) peak of the BCC. Another difficulty is lied in distinguishing the contributions of FCC and L1_2_ to the (200) fundamental peak. Here we assume that (1) the L1_2_ phases is of perfect order, and (2) the contributions of the FCC and L1_2_ to the peak intensity depend on only their contents. Marking FCC, L1_2_, and BCC as subscripts 1, 2, and 3, respectively, there is:

1 $$\left\{ \begin{array}{l}\frac{{I_2^{100}}}{{I_{1 + 2}^{200}}} = \frac{{C_2^{100}f_2}}{{C_1^{200}f_1 + C_2^{200}f_2}}\\ \frac{{I_3^{211}}}{{I_2^{100}}} = \frac{{C_3^{211}f_3}}{{C_2^{100}f_2}}\\ \mathop {\sum}\limits_{j = 1}^3 {f_j} = 1\end{array} \right.$$

where *I*_*i j*_ is the intensity of the *i* peak for the *j* phase, *f*_*j*_ is the fraction volume of the *j* phase, and *C*_*i j*_ is a parameter related to phase composition, Bragg angle, lattice structure, and phase nature. For synchrotron source, the absorptivity and Bragg angle are rather small, and hence *C*_*i j*_ can be evaluated according to the APT result. The volume fractions of the FCC, L1_2_, and BCC phases in 600 A were calculated to be 43.7%, 50.1%, and 6.2%, respectively. It should be noted that these values may not be absolutely precise, but they can serve as reasonable references for analysis of the strengthening mechanism in our ultrastrong PH-HEAs.

Detailed microstructure was characterized by a JEOL ARM200F spherical-aberration corrected transmission electron microscope (TEM), and atomic-resolution images were taken by a high-angle annular dark-field (HAADF) detector. The TEM specimens with dimensions of Ф3 mm × 0.5 mm were cut with a diamond wire saw and thinned to 40–60 μm by 3000- and 5000-grit SiC papers. After mechanically polishing, twin-jet electro-polishing was conducted using a mixed solution of HNO_3_:CH_3_OH = 1:4 at a temperature of ~235 K with a direct voltage of 28 V and a current of 55 mA. Final polishing was conducted on a Gatan 691 precision ion polishing system using a voltage of 2 kV for 7.5 min and a voltage of 1 kV for 15 min.

Atom probe tomography (APT) analysis for three-dimensional distribution of elements in materials was performed by a CAMECA local electrode atom probe LEAP^TM^ 4000X SI at a target specimen temperature of 20 K, under a pulsing UV laser with a pulse energy of 40 pJ, a pulse rate of 200 kHz, and an ion collection rate of 0.5% per pulse. A two-stage electro-polishing procedure was used to fabricate a sharp tip specimen (apex radius <50 nm) for APT analyses. The first stage electro-polishing was performed using an electrolyte containing 25 volume per cent (vol.%) perchloric acid in acetic acid at 15 V and room temperature, and the second stage was by a 2 vol.% perchloric acid in 2-butoxyethanol solution at 20 V. Reconstruction and quantitative analysis of the APT data were conducted on a CAMECA IVAS version 3.6.8 software. The compositions of the FCC, L1_2_ and BCC phases were estimated by averaging the core concentrations of the proximity histograms produced by reconstructing 22% Cr, 63% Ni, and 50% Cr (in at%) isoconcentration surfaces, respectively.

### Estimation of strengthening by various mechanisms

For near-equiatomic HEAs, strengthening by solute atoms is difficult to evaluate because distinguishing between the solvent and solute is difficult. Fortunately, solution strengthening should be similar for a given system, and hence the difference in the strength of ST alloys is attributed to various grain sizes. The intercept term *σ*_0_ in the Hall–Petch equation (Δ*σ*_gs_ = *σ*_0_ + *k*_g_∙*d*^−0.5^, where *k*_g_ is the Hall–Petch coefficient and *d* is the mean grain size)^21^ can be considered an alloy constant composed of solution strengthening Δ*σ*_ss_ and lattice friction stress *σ*_fs_. This consideration can reasonably simplify the estimation of the strengthening effect in multi-principal-element HEAs. To obtain different grain sizes, we annealed the as-rolled samples at 1200°C for various hold times (10, 30, and 120 min), and measured the grain sizes and tensile properties of these as-annealed samples. Using Hall–Petch equation to fit the experimental results (Supplementary Fig. 2), *σ*_0_ and *k*_g_ were determined to be 270.0 MPa and 378.6 MPa∙μm^0.5^, respectively, where *σ*_0_ = *σ*_fs_ + Δ*σ*_ss_. The mean grain sizes of 600 A and 700 A are 2.82 ± 0.77 and 1.62 ± 0.53 μm, respectively. The ± values mean the standard deviation given by EBSD. The contributions of grain boundaries to the yield strengths of the two samples are 212 and 284 MPa, respectively. Since recovery and recrystallization have been completed in all the samples after the final heat treatment, dislocation strengthening is negligible.

For spinodal structure, dislocations will be trapped by strong diffuse obstacles. The equation for evaluation of strengthening by spinodal structure is^19^:

2 $$\Delta \sigma _{{\mathrm{sds}}} = Mk_{\mathrm{s}}\left( {A\eta Y} \right)^{5/3}\left( {\lambda /Gb} \right)^{2/3}$$

where *M* = 3.06 is the Taylor factor, *k*_s_ = 0.122∙[*π*(1 − *υ*)/(1 − 2*υ*)]^2/3^ is a material constant, *υ* = 0.31 is the Poisson ratio, *A* = (*C* − *C*_0_)/3 is the amplitude of that modulation (in at%), *λ* is the wavelength of the composition modulation, and *η* = *a*^−1^∙*da*/*dC* is the lattice strains produced by spinodal decomposition, in which *a* is the lattice constant for the undecomposed sample, *da*/*dC* is the variation in the lattice constant with the composition *C*. According to the HEXRD data, the lattice constants of the undecomposed ST sample and FCC matrix in 600 A are 0.3596 and 0.3582 nm, respectively. Since the current system (600 A sample) is isotropic, *E* = 162 GPa is the elastic modulus, *Y* = *E*/(1 − *υ*) = 234.8 GPa is an elastic-dependent parameter, *G* = 61.8 GPa is the shear modulus, and *b* = $\sqrt 2 a/2$ is the Burgers vector. According to Supplementary Fig. 4b, *λ* is evaluated to be ~40 nm, whereas this value approximates to 70 nm (2*d*) based on the HEXRD result. However, the value evaluated based on the APT data is quite local while that calculated by the Scherrer equation is based on an assumption of spherical particles. Therefore, we use a *λ* value of 60 nm for calculation. In the current multi-principal-element system, it is difficult to evaluate the value of *A*. We observe that Fe and Cr exhibit similar cluster trends, and the content of Ni + Al + V is greater than 60 at% according to the APT data. We therefore assume that Ni + Al + V is the solvent for simplification. After ageing 1 h at 600°C, the solute concentrations change from 39.34 at% in the ST sample to 54.57 at% in the FCC matrix (rich region) based on the APT data. Accordingly, *A* = 5.1 at% and Δ*σ*_sds_ is calculated to be 406 MPa.

Strengthening by ordered coherent precipitates Δ*σ*_os_ occurs when dislocations shear these ordered phases and create APBs on the slip planes of them. We find that the distances between two coupled dislocations (~20–40 nm, as shown in Supplementary Fig. 6d) approximate to the mean diameter of the ordered L1_2_ phases (~30 nm), suggesting that the coupling of the dislocation in the pairs should be moderate (between strong and weak), and that the alloy should be aged to near the peak strength condition. Therefore, the YS increase Δ*σ*_os_ is given by^19^:

3 $$\Delta \sigma _{{\mathrm{os}}} = M0.81\frac{{\gamma _{{\mathrm{APB}}}}}{{2b}}\left( {\frac{{3\pi f}}{8}} \right)^{1/2}$$

in which *f* is the volume fraction of the ordered precipitates, *r* is the average radius of these ordered phases, and *γ*_APB_ = 0.2 J m^−2^ is the APB energy that is adopted from the data of Ni_3_Al precipitates^50^. Assuming that the L1_2_ precipitates are near spherical, *f* ≈ 50% and *r* is assumed to be 1/4*λ* (~15 nm) in 600 A; hence Δ*σ*_os_ is calculated to be 752 MPa. It should be noted that in our PH-HEAs the ordered L1_2_ nanoprecipitates are irregular and interconnected rather than spherical. The increases in YS calculated based on independent spheres is therefore not absolutely accurate and may underestimate the area of APBs and the strengthening effect. Nevertheless, the results can offer reasonable references to analyze the strengthening caused by the current spinodal order–disorder nanostructure.

Considering the BCC phases as the precipitates within FCC + L1_2_ matrix, we find that dislocations can shear and pass through these precipitates (Supplementary Fig. 6a–c). It is noteworthy that two orientation relationships, i.e. $[\bar 111]_{{\mathrm{BCC}}}//[{\mathrm{0}}11]_{{\mathrm{FCC}}}$ and $(110)_{{\mathrm{BCC}}}//(11\bar 1)_{{\mathrm{FCC}}}$, exist between the BCC phases and matrix (Supplementary Fig. 7). These observations mean that these BCC phases share the slide planes with the matrix, and that the specific energy of new interfaces created by shearing is low, leading to weak chemical strengthening. Moreover, elastic lattice strain induced by semi-coherent precipitate–matrix interfaces is low enough to be negligible. Instead, the difference in shear moduli between the BCC precipitates and matrix is high (Δ*G* = *G*_BCC_–*G*_matrix_ = 100–85 *=* 15 GPa, where *G*_BCC_ is adopted from the data of a Cr−22 at% Fe alloy^51,52^ and *G*_matrix_ is a value selected between the shear modulus of CoCrFeNi^17^ and that of CoCrNi^16^). Therefore, modulus strengthening Δ*σ*_ms_, which should be the primary contribution to the strength increase, is given by^19^:

4 $$\Delta \sigma _{{\mathrm{ms}}} = M0.0055\left( {\Delta G} \right)^{\frac{3}{2}}\left( {\frac{{2f}}{G}} \right)^{\frac{1}{2}}\left( {\frac{r}{b}} \right)^{\frac{{3m}}{2} - 1}$$

where *m* is a constant and is equal to 0.85 for FCC-based alloys. In the 600 A sample, the BCC phases are plate-shaped with dimensions of ~500 × 50 × 10 nm^3^. For simplification, we assume the BCC phases to be spherical with diameters of 50 nm because the projected lengths of the BCC phases at the slide planes approximate to this value. Accordingly, Δ*σ*_ms_ is calculated to be 154 MPa. It is worth pointing out that this assumption significantly increases the number density of the BCC phases for a fixed *f*, leading to overestimation of the contribution of the BCC phases to the yield strength. Nevertheless, strengthening by the BCC precipitates is confirmed to be low, and the calculated result agrees well with the actual increases in strength.

## Electronic supplementary material

Supplementary Information

## Data Availability

The data that support the findings of this study are available from the corresponding author on reasonable request.

## References

[1] Yeh JW (2004). Nanostructured high-entropy alloys with multiple principal element: novel alloy design concepts and outcomes. Adv. Eng. Mater..

[2] Zhang Y (2014). Microstructures and properties of high-entropy alloys. Prog. Mater. Sci..

[3] Pickering EJ, Jones NG (2016). High-entropy alloys: a critical assessment of their founding principles and future prospects. Int. Mater. Rev..

[4] Gludovatz B (2014). A fracture-resistant high-entropy alloy for cryogenic applications. Science.

[5] Liu WH, Yang T, Liu CT (2018). Precipitation hardening in CoCrFeNi-based high entropy alloys. Mater. Chem. Phys..

[6] Miracle DB, Senkov ON (2016). A critical review of high entropy alloys and related concepts. Acta Mater..

[7] Liu WH (2016). Ductile CoCrFeNiMo_X_ high entropy alloys strengthened by hard intermetallic phases. Acta Mater..

[8] Ming K, Bi X, Wang J (2017). Precipitation strengthening of ductile Cr_15_Fe_20_Co_35_Ni_20_Mo_10_ alloys. Scr. Mater..

[9] Wani IS (2016). Tailoring nanostructures and mechanical properties of AlCoCrFeNi_2.1_ eutectic high entropy alloy using thermo-mechanical processing. Mater. Sci. Eng. A.

[10] Li Z, Pradeep KG, Deng Y, Raabe D, Tasan CC (2016). Metastable high-entropy dual-phase alloys overcome the strength-ductility trade-off. Nature.

[11] Lu Y (2017). Directly cast bulk eutectic and near-eutectic high entropy alloys with balanced strength and ductility in a wide temperature range. Acta Mater..

[12] Huang H (2017). Phase-transformation ductilization of brittle high-entropy alloys via metastability engineering. Adv. Mater..

[13] Li D (2017). High-entropy Al_0.3_CoCrFeNi alloy fibers with high tensile strength and ductility at ambient and cryogenic temperatures. Acta Mater..

[14] Gwalani B (2017). Optimizing the coupled effects of Hall-Petch and precipitation strengthening in a Al_0.3_CoCrFeNi high entropy alloy. Mater. Des..

[15] Wang ZG (2017). Effect of coherent L1_2_ nanoprecipitates on the tensile behavior of a fcc-based high-entropy alloy. Mater. Sci. Eng. A.

[16] Zhao YL (2017). Heterogeneous precipitation behavior and stacking-fault-mediated deformation in a CoCrNi-based medium-entropy alloy. Acta Mater..

[17] He JY (2016). A precipitation-hardened high-entropy alloy with outstanding tensile properties. Acta Mater..

[18] Liu CT, Stiegler JO (1984). Ductile ordered intermetallic alloys. Science.

[19] Ardell AJ (1985). Precipitation hardening. Metall. Trans. A.

[20] Reed, R. C. *The Superalloys: Fundamentals and Applications*. (Cambridge university press, New York, 2006).

[21] Courtney, T. H. *Mechanical Behavior of Materials*. Second edn. (McGraw-Hill, Boston, 2000).

[22] Cahn JW (1965). Phase separation by spinodal decomposition in isotropic systems. J. Chem. Phys..

[23] Viswanathan GB (2011). Precipitation of ordered phases in metallic solid solutions: a synergistic clustering and ordering process. Scr. Mater..

[24] Booth-Morrison C (2008). Effects of solute concentrations on kinetic pathways in Ni–Al–Cr alloys. Acta Mater..

[25] Lu K (2016). Stabilizing nanostructures in metals using grain and twin boundary architectures. Nat. Rev. Mater..

[26] Jiang S (2017). Ultrastrong steel via minimal lattice misfit and high-density nanoprecipitation. Nature.

[27] Lenkkeri JT, Lahteenkorva EE (1978). An investigation of elastic moduli of vanadium-chromium alloys. J. Phys. F Metal Phys..

[28] Singh S, Wanderka N, Murty BS, Glatzel U, Banhart J (2011). Decomposition in multi-component AlCoCrCuFeNi high-entropy alloy. Acta Mater..

[29] Santodonato LJ (2015). Deviation from high-entropy configurations in the atomic distributions of a multi-principal-element alloy. Nat. Commun..

[30] Otto F (2016). Decomposition of the single-phase high-entropy alloy CrMnFeCoNi after prolonged anneals at intermediate temperatures. Acta Mater..

[31] Wen LH (2009). Effect of aging temperature on microstructure and properties of AlCoCrCuFeNi high-entropy alloy. Intermetallics.

[32] Singh S, Wanderka N, Kiefer K, Siemensmeyer K, Banhart J (2011). Effect of decomposition of the Cr-Fe-Co rich phase of AlCoCrCuFeNi high entropy alloy on magnetic properties. Ultramicroscopy.

[33] Wang WR (2012). Effects of Al addition on the microstructure and mechanical property of Al_X_CoCrFeNi high-entropy alloys. Intermetallics.

[34] Pickering EJ, Stone HJ, Jones NG (2015). Fine-scale precipitation in the high-entropy alloy Al_0.5_CrFeCoNiCu. Mater. Sci. Eng. A.

[35] Tong CJ (2005). Mechanical performance of the Al_X_CoCrCuFeNi high-entropy alloy system with multiprincipal elements. Metall. Mater. Trans. A.

[36] Tong CJ (2005). Microstructure characterization of Al_X_CoCrCuFeNi high-entropy alloy system with multiprincipal elements. Metall. Mater. Trans. A.

[37] Tung CC (2007). On the elemental effect of AlCoCrCuFeNi high-entropy alloy system. Mater. Lett..

[38] Manzoni A, Daoud H, Volkl R, Glatzel U, Wanderka N (2013). Phase separation in equiatomic AlCoCrFeNi high-entropy alloy. Ultramicroscopy.

[39] Lu K, Lu L, Suresh S (2009). Strengthening materials by engineering coherent internal boundaries at the nanoscale. Science.

[40] Lu L, Chen X, Huang X, Lu K (2009). Revealing the maximum strength in nanotwinned copper. Science.

[41] Li X, Wei Y, Lu L, Lu K, Gao H (2010). Dislocation nucleation governed softening and maximum strength in nano-twinned metals. Nature.

[42] Hanna JA, Baker I, Wittmann MW, Munroe PR (2011). A new high-strength spinodal alloy. J. Mater. Res..

[43] Xia S, Yang X, Chen M, Yang T, Zhang Y (2017). The Al effects of Co-free and V-containing high-entropy alloys. Metals.

[44] Liu S, Gao MC, Liaw PK, Zhang Y (2015). Microstructures and mechanical properties of Al_X_CrFeNiTi_0.25_ alloys. J. Alloy Compd..

[45] Laktionova MA, Tabchnikova ED, Tang Z, Liaw PK (2013). Mechanical properties of the high-entropy alloy Ag_0.5_CoCrCuFeNi at temperatures of 4.2–300 K. Low Temp. Phys..

[46] Nelson JB, Riley DP (1945). An experimental investigation of extrapolation methods in the derivation of accurate unit-cell dimensions of crystals. Proc. Phys. Soc..

[47] Patterson AL (1939). The scherrer formula for X-ray particle size determination. Phys. Rev..

[48] Langford JI, Wilson AJC (1978). Seherrer after sixty years: a survey and some new results in the determination of crystallite size. J. Appl. Crystallogr..

[49] Ungár T (2004). Microstructural parameters from X-ray diffraction peak broadening. Scr. Mater..

[50] Kozar RW (2009). Strengthening mechanisms in polycrystalline multimodal nickel-base superalloys. Metall. Mater. Trans. A.

[51] Lenkkeri JT (1980). The elastic moduli of some body-centred cubic titanium-vanadium, vanadium-chromium and chromium-iron alloys. J. Phys. F Metal. Phys..

[52] Razumovskiy VI, Ruban AV, Korzhavyi PA (2011). First-principles study of elastic properties of Cr- and Fe-rich Fe-Cr alloys. Phys. Rev. B.

