# Supplementary material for: High-content ductile coherent nanoprecipitates achieve ultrastrong high-entropy alloys
Source: Nat Commun. 2018 Oct 3;9:4063. doi: 10.1038/s41467-018-06600-8 (PMC6170471; doi:10.1038/s41467-018-06600-8)
Supplement: Supplementary file 1 — Supplementary Information [file 41467_2018_6600_MOESM1_ESM.pdf]

Supplementary Information

High-content ductile coherent nanoprecipitates achieve ultrastrong high-entropy alloys

Liang et al.

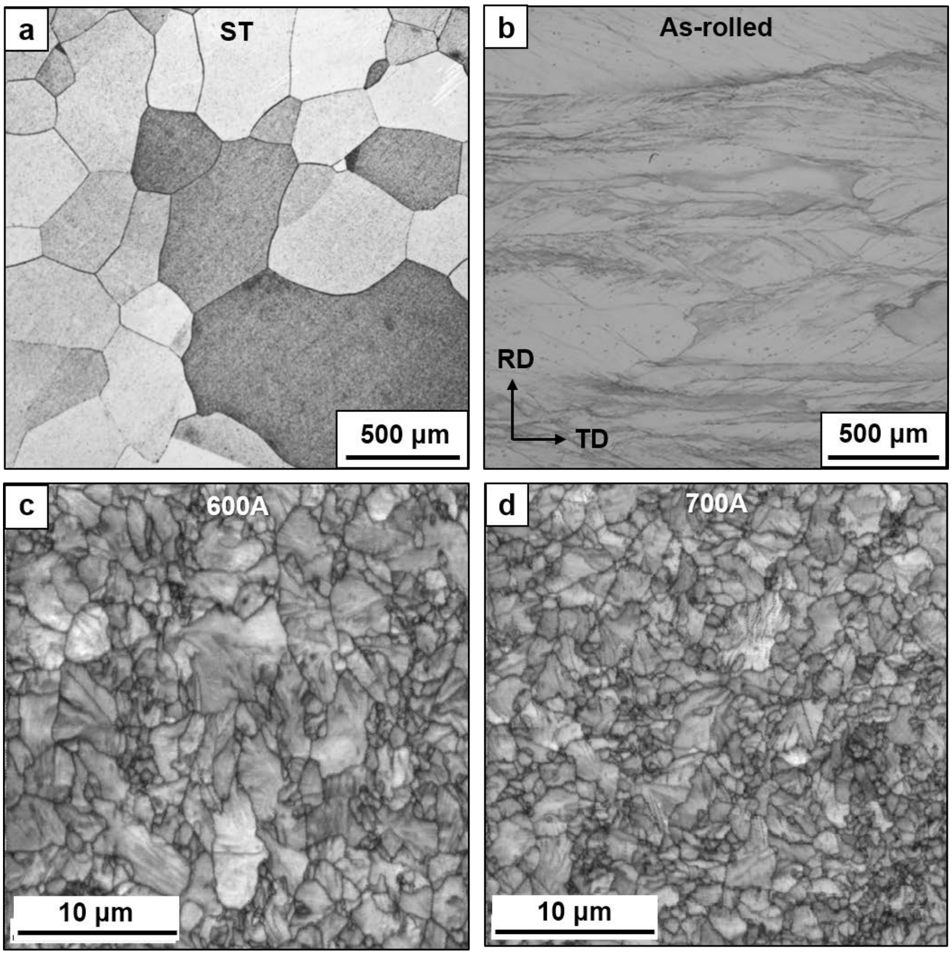

Supplementary Figure 1 | Grain morphologies of the current HEA produced under various processing conditions. a, Homogenized and coarse equiaxed FCC grains forming after solution treatment. **b,** FCC grains were elongated after 72% cold-rolling. **c,d,** Fine recrystallization grains obtained by ageing for 1 h at 600°C and 700°C after cold-rolling, respectively.

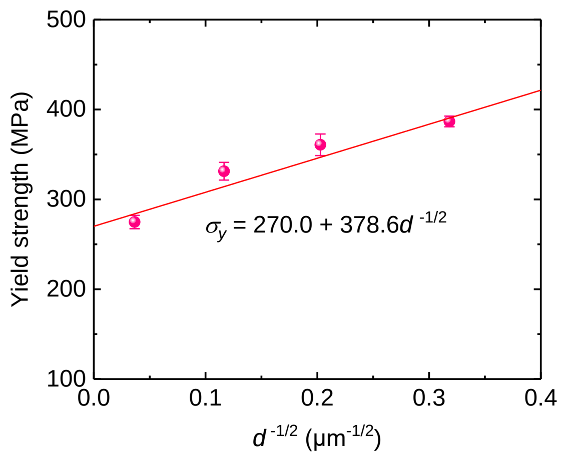

Supplementary Figure 2 | Strengthening by lattice friction stress, solute atoms, and grain boundaries. For a fixed alloy, the intercept term σ_0 in the Hall–Petch equation ($\sigma_y = \sigma_0 + k_g \cdot d^{-0.5}$, where k_g is the Hall–Petch coefficient) is considered an alloy constant composed of solution strengthening $\Delta\sigma_{ss}$ and lattice friction stress σ_{fs}. Linear fitting of the plot of the YS σ_y of single-phase FCC alloys versus the inverse of the square root of grain size d with the Hall–Petch equation can estimate the strengthening effects of these mechanisms experimentally. Error bars, s.d..

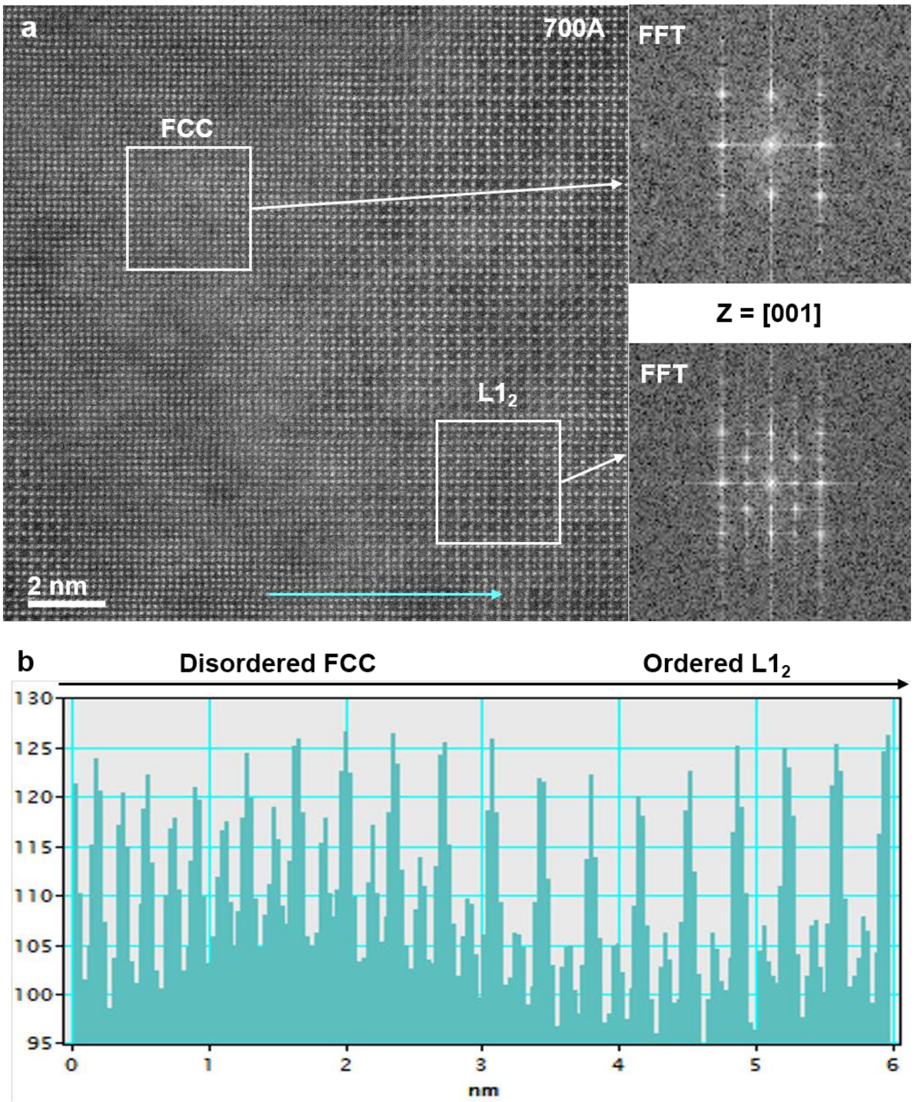

Supplementary Figure 3 | Structure of the phases existing in 700A. a, Atomic mass contrast in atomic-resolution HAADF-STEM images of 700A showing the nanostructure consisting of a disordered FCC matrix and ordered $L1_2$ phases with diffuse coherent interfaces. **b,** Intensity profile along the cyan arrow marked in **a** showing atomic arrangement in the two phases.

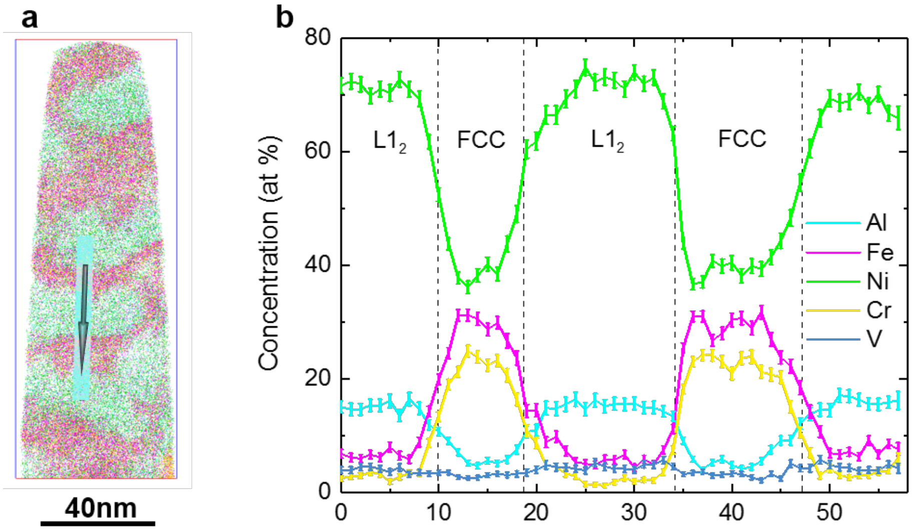

c

	Al	Cr	Fe	Ni	V
**Disordered FCC**	5.08	25.01	29.56	37.51	2.84
**Ordered L1$_2$**	18.17	2.53	6.49	68.04	4.77

Supplementary Figure 4 | Atom probe analysis of the element distribution and composition in the spinodal order-disorder nanostructure. a, Three-dimensional reconstruction of atomic distributions in a thin slice with a thickness of 10 nm. **b,** One-dimensional concentration profiles (sampling region and direction marked in **a**) demonstrating long-range periodic composition fluctuations without abrupt changes in composition. Error bars, s.d.. **c,** Mean compositions (in at%) of the disordered FCC matrix and ordered L1$_2$ nanoprecipitates.

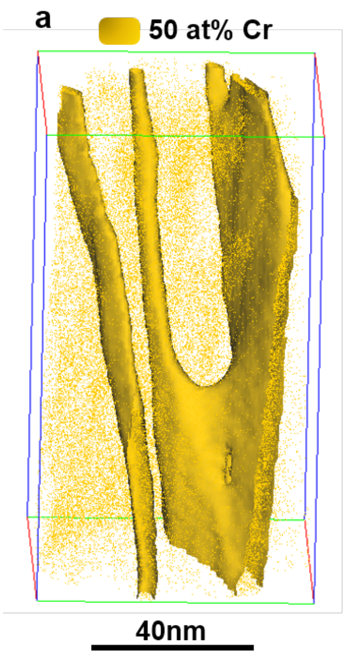

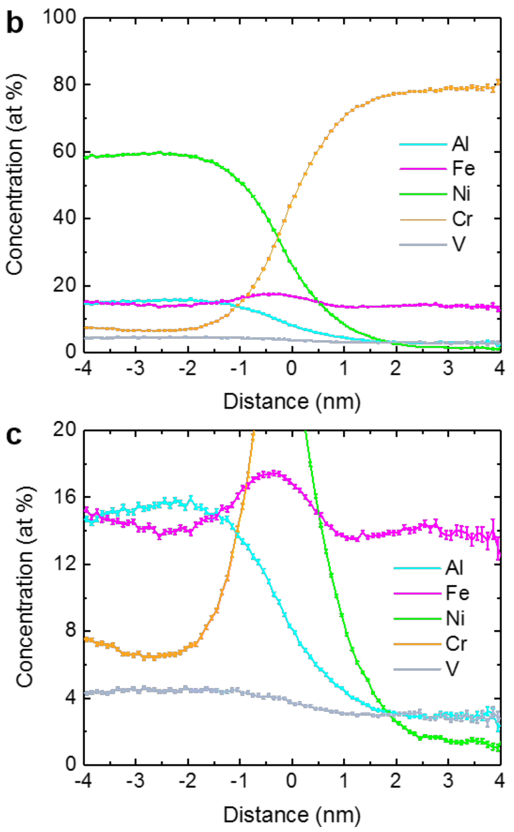

d

Al	Cr	Fe	Ni	V
2.93	78.86	13.78	1.60	2.85

Supplementary Figure 5 | Atom probe analysis of the composition and morphology of the BCC phases. a, Three-dimensional reconstruction of 50 at% Cr isoconcentration surfaces showing the morphology of the BCC phases. **b,** Proximity histogram presenting the element distributions in vicinity of the 50 at% Cr isoconcentration surfaces. Error bars, s.d.. **c,** Corresponding close-up histogram of **b** revealing that Fe element is enriches at the interfaces. Error bars, s.d.. **d,** Mean composition (in at%) of the BCC phases.

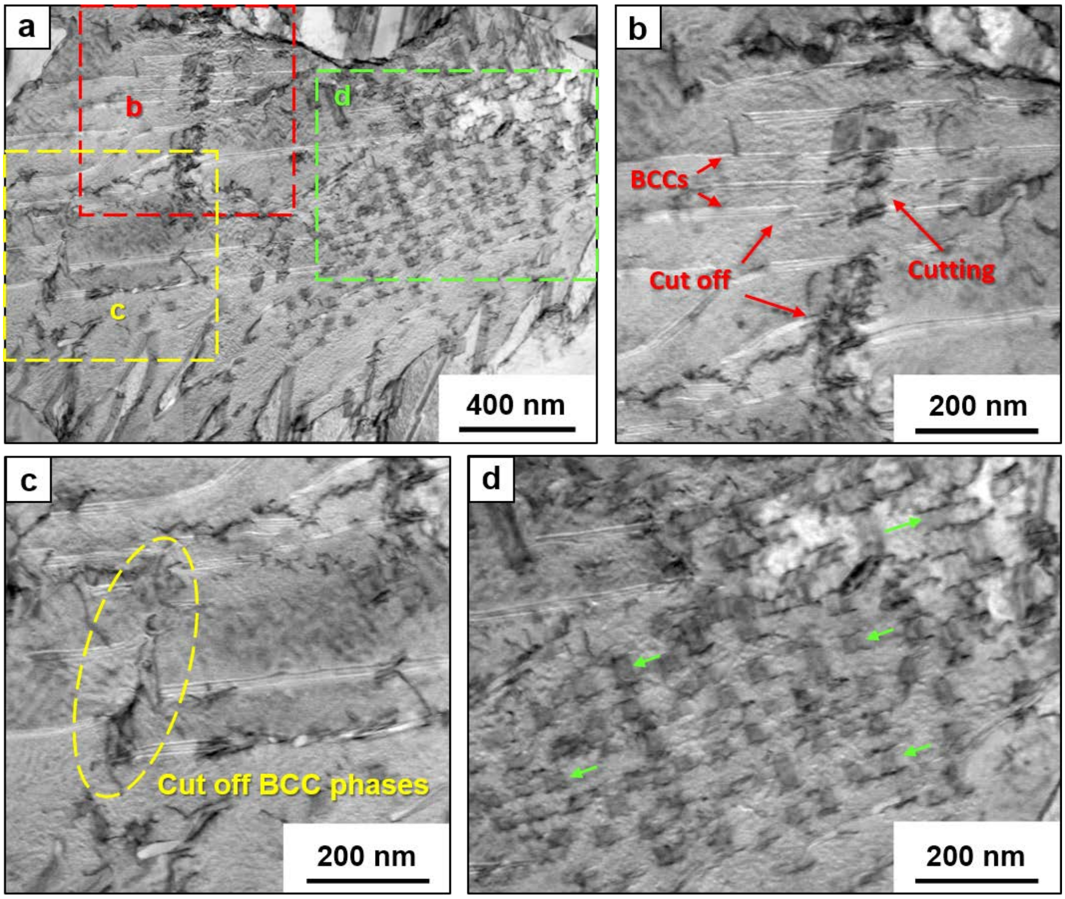

Supplementary Figure 6 | Bright field (BF) STEM images of the 600A sample after tensile deformation. a, Interaction between the precipitates and dislocations. **b,c,** High-magnification images revealing that dislocations shear and pass through the BCC phases. **d,** Numerous coupled dislocations existing in FCC + $L1_2$ region.

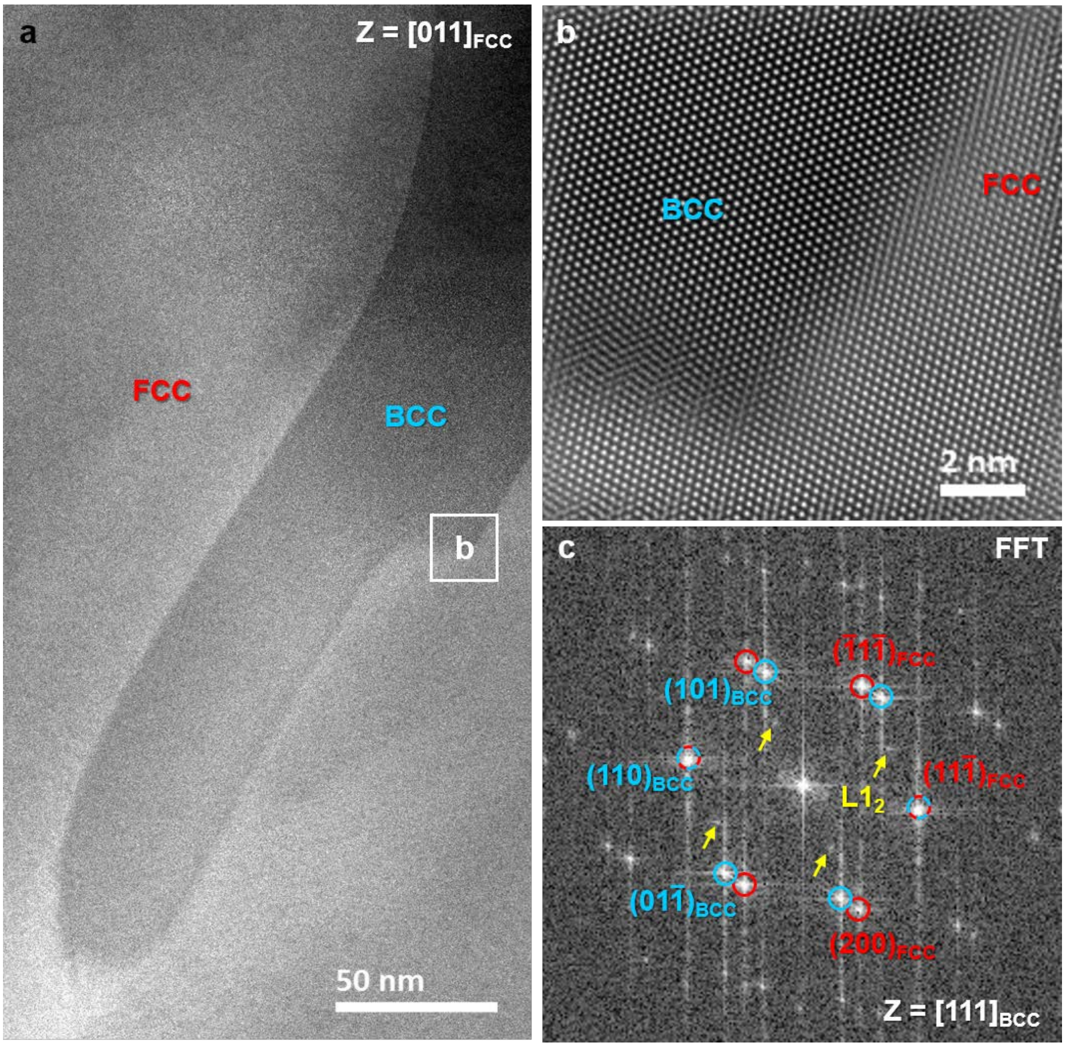

Supplementary Figure 7 | Interfacial structure and orientation relationship between the BCC precipitates and FCC+L1$_2$ matrix. a, HAADF-STEM images showing the morphology of a BCC phase. **b,** Atomic-resolution HAADF-STEM images of the region marked by the white box in **a** showing a semi-coherent precipitate–matrix interface. **c,** FFT of **b** showing that two orientation relationships, $[\bar{1}11]_{BCC}//[011]_{FCC}$ and $(110)_{BCC}//(11\bar{1})_{FCC}$, exist between the precipitate and matrix.

Supplementary Table 1 | Compositions, processing, microstructure, and tensile properties of the HEAs studied previously.

Systems	Alloys	Processing [a]	Microstructure [a]	$\dot{\varepsilon}$ (s^{-1})	σ_y (MPa)	σ_{uts} (MPa)	ε (%)	Ref.
AlCoCrCuFeNi	AlCoCrCuFeNi	AC	BCC+2FCC	1×10^{-3}	790	790	0.2	1
		AC + 960°C/50h + multi-step forged at 950°C (1000%)	BCC+2FCC+σ	1×10^{-3}	1040	1170	1	1
	$Al_{0.5}CoCrCuFeNi$	AC	2FCC	8×10^{-4}	359	707	19	2
		AC	2FCC	1×10^{-3}	578.7	895	10.7	3
		AC + 600°C/24h/WQ	FCC+BCC	1×10^{-3}	663.8	1002	8.1	3
		AC + 1000°C/6h/WQ + CR (80%)	2FCC	1×10^{-3}	1292	1406	6	4
		AC + 1000°C/6h/WQ + CR (80%)	2FCC	1×10^{-3}	1284	1344	7.6	5
		AC + 1000°C/6h/WQ + CR (84%)	FCC + $L1_2$	1×10^{-3}	1284	1344	7.6	6
		AC + 1000°C/6h/WQ + CR (80%) + 900°C/10min	2FCC	1×10^{-3}	1021	1030 [b]	15.3	5
		AC + 1000°C/6h/WQ + CR (80%) + 900°C/30min	2FCC	1×10^{-3}	970 [b]	980 [b]	18 [b]	5
		AC + 1000°C/6h/WQ + CR (80%) + 900°C/60min	2FCC	1×10^{-3}	810 [b]	870 [b]	23 [b]	5
		AC + 1000°C/6h/WQ + CR (80%) + 900°C/300min	2FCC	1×10^{-3}	610	780 [b]	28 [b]	5
		AC + 1000°C/6h/WQ + CR (80%) + 900°C/5h	2FCC, GS = 1 μm	1×10^{-3}	656	796	29	4
		AC (with EMS)	FCC	2.5×10^{-4}	570 [b]	693.66	25	7
		AC (without EMS)	FCC	2.5×10^{-4}	520 [b]	616.76	30	7
	$Al_{0.2}CoCrCu_{0.2}FeNi_2$	AC + 1200°C/24h + CR (93%) + 700°C/20h/WQ	FCC+$L1_2$, GS = 4.49μm	1.7×10^{-3}	719	1048	30.4	8
		AC + 1200°C/24h + CR (93%) + 800°C/1h/WQ	FCC, GS = 4.51μm	1.7×10^{-3}	460	732	31.7	8
	$Al_{0.5}CoCrCu_{0.5}FeNi_2$	AC	FCC + $L1_2$	3.3×10^{-3}	357	459	9	9
		AC + 700°C/5h/SC	FCC+$L1_2$+Cr-rich IM at GB	3.3×10^{-3}	365	365	0.1	9
		AC + 1150°C/5h/WQ	FCC + $L1_2$	3.3×10^{-3}	215	489	39	9
AlCoCrFeNi	$Al_{0.1}CoCrFeNi$	As-received	FCC, GS = few mm	1×10^{-3}	160±7	389±42	44±14.5	10
		FSP	FCC, GS = 0.35-13.5μm	1×10^{-3}	544±50	730±19	27.5±1.4	10
		As-received	FCC, GS = few mm	1×10^{-3}	150±1	320 [b]	50 [b]	11
		FSP	FCC, GS = 14±10μm	1×10^{-3}	315±4	640 [b]	75	11
	$Al_{0.25}CoCrFeNi$	AC	FCC	5×10^{-4}	118±4.0	807±75.5	55.2±0.9	12
		AC + CR (88.5%) + 1000°C/10h/SC	FCC	5×10^{-4}	48±8.5	271±25.6	20.7±1.4	12
		AC + CR (80%) + 1000°C/10h/SC	FCC	5×10^{-4}	72±7.4	556±46.0	34.9±1.0	12
		AC + CR (73%) + 1000°C/10h/SC	FCC	5×10^{-4}	119±0.7	697±8.7	47.9±1.7	12

		AC + CR (60%) + 1000°C/10h/SC	FCC	5×10^{-4}	126±4.5	734±30.0	50.4±0.9	12
		AC + CR (50%) + 1000°C/10h/SC	FCC	5×10^{-4}	150±5.0	758±34.0	51.1±1.3	12
		AC + CR (25%) + 1000°C/10h/SC	FCC	5×10^{-4}	139±22.7	805±30.9	46.8±1.74	12
	$Al_{0.3}CoCrFeNi$	AC	$FCC+L1_2$	4×10^{-4}	170 [b]	340 [b]	60 [b]	13
		AC	FCC	4×10^{-4}	275	528	37	14
		AC (DS)	FCC, Single-crystal near [001]	4×10^{-4}	185	399	80	14
		Direct laser fabrication	FCC	1×10^{-3}	194	270 [b]	40 [b]	15
		AC + 700°C/72h/WQ	$FCC+L1_2$	4×10^{-4}	310 [b]	525 [b]	44 [b]	13
		AC + 900°C/72h/WQ	FCC+B2	4×10^{-4}	240 [b]	570 [b]	45 [b]	13
		AC + CR (90%) + 1150°C/2min	$FCC+L1_2+B2$	1×10^{-3}	263±32	589±51	60±7	16
		AC + CR (90%) + 1150°C/2min + 620°C/50h	$FCC+L1_2+B2$	1×10^{-3}	490±22	840±24	45±8	16
		AC + CR (90%) + 1150°C/5min	$FCC+L1_2+B2$	1×10^{-3}	220±20	550±35	60±4	16
		AC + CR (90%) + 1150°C/60min	$FCC+L1_2+B2$	1×10^{-3}	159±22	410±42	65±3	16
		AC + CR (90%) + 1150°C/60min, 700°C/50h	$FCC+L1_2+B2$	1×10^{-3}	215±16	520±21	43±6	16
		AC + CR (90%) + 1150°C/60min, 550°C/150h	$FCC+L1_2+B2$	1×10^{-3}	285±14	540±43	55±12	16
		AC + 1250°C/50h/SC + 1250°C UF (50%)	FCC	2×10^{-4}	210	500	97	17
		AC + 1050°C HF + 1000°C RS + 900°C HD	FCC+B2, Φ1mm fiber	2×10^{-4}	1136	1207	7.8	18
	$Al_{0.5}CoCrFeNi$	AC	FCC+BCC	1×10^{-3}	355	714	41.6	19
		AC + 650°C/8 h/WQ	FCC+BCC+B2	1×10^{-3}	834	1220	25	19
		AC + 1250°C/50h/SC + 1250°C UF (50%)	FCC+BCC/B2	2×10^{-4}	550	725	56	17
	$Al_{0.7}CoCrFeNi$	AC + 1250°C/50h/SC + 1250°C UF (50%)	FCC+BCC/B2	2×10^{-4}	600	740	8	17
	$AlCoCrFeNi_{2.0}$	AC	FCC+B2	1×10^{-3}	545	1070 [b]	17 [b]	20
	$AlCoCrFeNi_{2.1}$	AC	FCC+B2	1×10^{-3}	545	1040 [b]	18 [b]	20
		AC	FCC+B2	1×10^{-3}	75	944	25.6	21
		AC	$L1_2+B2$	8.3×10^{-4}	620	1050	17	22,23
		AC + CR (8%)	FCC+B2	2×10^{-3}	275	1145	10.4	21
		AC + CR (90%)	FCC+B2	8.3×10^{-4}	1625	1800	6	22,23
		AC + CR (90%) + 800°C/1h	FCC+B2	8.3×10^{-4}	1108	1200	12	22,23
		AC + CR (90%) + 1000°C/1h	FCC+B2	8.3×10^{-4}	844	1175	23	23
		AC + CR (90%) + 1200°C/1h	FCC+B2	8.3×10^{-4}	648	1075	27	23
	$AlCoCrFeNi_{2.2}$	AC	FCC+B2	1×10^{-3}	545	1120 [b]	20.5 [b]	20
	$Al_{0.7}CoCrFe_2Ni$	AC	FCC+BCC+B2	2×10^{-4}	866	1223	7.9	24

AlCoCrNiTi	$Al_{0.096}CoCrNiTi_{0.096}$	AC + 1200°C/2h/WQ + CR (66%) + 1160°C/3min/WQ + 800°C/2h/WQ	FCC+$L1_2$, GS = 67μm	1×10^{-3}	750	1260 [b]	45	25
AlCoCrFeNiTi	$Al_{0.17}CoCrFeNiTi_{0.09}$	AC + 1200°C/4h	FCC	1×10^{-3}	185 [b]	503	67 [b]	26
		AC + 1200°C/4h + CR (30%) + 1000°C/2h + 800°C/18h/WQ	FCC+$L1_2$+Ni_2AlTi	1×10^{-3}	645	1094	39	26
		AC + 1200°C/4h + CR (70%) + 650°C/4h/WQ	FCC+$L1_2$+Ni_2AlTi	1×10^{-3}	1005	1273	17	26
AlCoFeNiSi	$Al_{0.2}CoFeNiSi_{0.2}$	AC	FCC	5×10^{-4}	280	632	41.6	27
		AC + CR (60%)	FCC	5×10^{-4}	1149	1149	4	27
AlCoCrFeMnNi	$Al_{0.4}CoCrFeMnNi$	AC	FCC	1×10^{-3}	242	529	47.2	28
	$Al_{0.6}CoCrFeMnNi$	AC	FCC+BCC+B2	1×10^{-3}	832	1174	7.7	28
AlCrCuFeNi	$Al_{0.5}CrCuFeNi_2$	AC + CR (43%)	2FCC	1×10^{-3}	363±60	500±20	16±7	29
		AC + CR (43%) + 700°C/24 h	BCC+FCC+ $L1_2$	1×10^{-3}	630±270	922±240	4.2±1.3	29
		AC + CR (43%) + 900°C/24 h	BCC+FCC+ $L1_2$	1×10^{-3}	704±180	1088±20	5.6±3.2	29
		AC + CR (43%) + 1100°C/24 h	FCC+ $L1_2$	1×10^{-3}	360±100	639±5	3.4±0.4	29
	$Al_{0.5}CrCuFeNi_2$	AC	FCC	4×10^{-4}	390	470	-	30
		AC + CR (50%)	FCC	4×10^{-4}	1055	1179	2	30
AlCrFeNi	$AlCrFe_2Ni_2$	AC	FCC+BCC+B2	1×10^{-3}	796	1437	15.7	31
AlCrFeMnNi	$Al_{0.6}CrFe_2Mn_{1.2}Ni_{0.8}$	AC	BCC+B2	1×10^{-3}	750	880	2.5	32
	$Al_{0.7}Cr_{0.5}Fe_{3.6}Mn_{3.1}Ni$	AC	FCC, GS = 123μm	5×10^{-4}	170 [b]	375 [b]	40 [b]	33
		AC + CR (70%) + 800°C/8h	FCC+B2, GS = 5μm	5×10^{-4}	416	530 [b]	30 [b]	33
		AC + CR (70%) + 800°C/30h	FCC+B2, GS = 7μm	5×10^{-4}	361	680 [b]	30 [b]	33
		AC + CR (70%) + 900°C/8h	FCC+B2, GS = 19μm	5×10^{-4}	219	700 [b]	43 [b]	33
	$Al_{0.7}Cr_{0.5}Fe_{3.6}NiMn_{3.1}C_{0.09}$	AC	FCC, GS = 118μm	5×10^{-4}	380 [b]	870 [b]	48 [b]	33
		AC + CR (70%) + 1000°C/1h	FCC+B2+$M_{23}C_6$+M_7C_3, GS = 8μm	5×10^{-4}	557	1050 [b]	26 [b]	33
		AC + CR (70%) + 1000°C/8h	FCC+B2+$M_{23}C_6$+M_7C_3, GS = 23μm	5×10^{-4}	488	950 [b]	28 [b]	33
		AC + CR (70%) + 1100°C/4h	FCC+B2+ M_7C_3, GS = 5μm	5×10^{-4}	405	910 [b]	38 [b]	33
AlFeMnNi	$Al_{0.7}Fe_2Mn_{1.8}Ni$	AC	FCC+B2	5×10^{-4}	270 [b]	580 [b]	23 [b]	34
		AC + 727°C/1h	FCC+B2	5×10^{-4}	420 [b]	780 [b]	22 [b]	34
		AC + 727°C/72h	FCC+B2	5×10^{-4}	483	880 [b]	15 [b]	34
	$Al_{0.7}Fe_2Mn_{1.8}NiC_{0.07}$	AC	FCC+MS	5×10^{-4}	260 [b]	680 [b]	39.3	34
		AC + 727°C/1h	FCC + MS + B2	5×10^{-4}	540 [b]	875 [b]	15 [b]	34
		AC + 727°C/72h	FCC + MS + B2	5×10^{-4}	611	940 [b]	10 [b]	34

		AC + CR (70%) + 1000°C/1h	FCC + MS + B2, GS = 1.9μm	5×10^{-4}	532	930 [b]	23.8	34
		AC + CR (70%) + 1100°C/4h + CR (50%) + 1000°C/4h	FCC + MS + B2, GS = 4.5μm	5×10^{-4}	426	945 [b]	32.8	34
CoCrFeNi	CoCrFeNi	AC	FCC	8.3×10^{-5}	188	457	50	35
		AC	FCC	1×10^{-3}	140	488	83	36
		AC	FCC	1×10^{-3}	147	413	48	37
		AC + 1000°C/24h	FCC	1×10^{-3}	130	458	87	36
		AC + 1200°C/4h	FCC	1×10^{-3}	165 [b]	453	67 [b]	26
		AC + 500°C/4h	FCC	1×10^{-3}	155	472.4	58.9	38
		AC + 1000°C/24h/SC + CR (80%) + 1100°C/1h/SC	FCC, GS = 60-80μm	7.3×10^{-4}	197	582	82.4	39
		AC + 1000°C/24h/SC + CR (80%) + 625°C/1h/SC	FCC, GS = 1-2μm	7.3×10^{-4}	540	786	49.3	39
		AC + 1000°C/24 h + 1000°C HR (92%) + 900°C/1h	FCC, GS = 11 μm	1×10^{-3}	300	671	42	40
		AC + 1200°C/24 h + CR (92%) + 900°C/1h	FCC, GS = 24μm	1×10^{-3}	273	714	38	41
		SLM/50μm per layer	FCC	8.3×10^{-5}	402	480	8	35
		SLM/20μm per layer	FCC	8.3×10^{-5}	600	745	32	35
		SLM/20μm per layer + 750°C/12h/WQ	FCC	8.3×10^{-5}	495	695	30	35
		SLM/20μm per layer + 1000°C/12h/WQ	FCC	8.3×10^{-5}	433	682	42	35
CoCrFeMn	$Co_{0.25}Cr_{0.25}FeMn$	AC + 900°C HR (50%) + 1200°C/2h/WQ	FCC	1×10^{-3}	240	489	58	42
	$Co_{0.2}Cr_{0.2}FeMn_{0.6}$	AC + 900°C HR (50%) + 1200°C/2h/WQ	FCC+HCP, GS = 45μm	1×10^{-3}	250	730	50	43
		AC + 900°C HR (50%) + 1200°C/2h/WQ + CR (60%) + 900°C/3min/WQ	FCC+HCP, GS = 4.5μm	1×10^{-3}	340 [b]	870	75	43
		AC + 900°C HR (50%) + 1200°C/2h/WQ + CR (60%) + 900°C/30min/WQ	FCC+HCP, GS = 15μm	1×10^{-3}	305	830	63	43
	$Co_{0.24}Cr_{0.24}FeMn_{1.2}$	AC + 900°C HR (50%) + 1200°C/2h/WQ	FCC, GS = 90μm	1×10^{-3}	100 [b]	360 [b]	47 [b]	44
	$Co_{0.2}Cr_{0.2}FeMn_{0.6}$	AC + 900°C HR (50%) + 1200°C/2h/WQ + CR (60%) + 900°C/3min/WQ	FCC+HCP, GS = 4.5μm	1×10^{-3}	340 [b]	870	75	44
CoCrMnNi	CoCrMnNi	AC + 1100°C/24 h + CR (90%) + 1000°C/1h	FCC, GS = 36μm	1×10^{-3}	280	699	43	41
CoFeMnNi	CoFeMnNi	AC + 1100°C/24 h + CR (90%) + 1000°C/1h	FCC, GS = 48μm	1×10^{-3}	175	551	41	41
CrFeMnNi	$Cr_{0.66}FeMnNi$	AC + 1200°C/24h/WQ + CR (86%) + 900°C/1h	FCC	1×10^{-3}	265 [b]	630 [b]	37 [b]	45
CoCrFeMnNi	CoCrFeMnNi	AC	FCC	1×10^{-3}	209	496	61.7	28
		AC	FCC	1×10^{-3}	215	491	71	36
		AC + 1000°C/24h	FCC	1×10^{-3}	162	443	68	36

		AC + 1000°C/24h/SC + CR (80%) + 1100°C/1h	FCC, GS = 60-80μm	7.3×10^{-4}	135	497	55.4	39
		AC + 1000°C/24h/SC + CR (80%) + 650°C/1h	FCC, GS = 1-2μm	7.3×10^{-4}	660	851	24.9	39
		AC + CF & CR (60%) + 800°C/1h	FCC, GS = 6 μm	1×10^{-3}	410±21	763±32	57±7	46
		AC + 1000°C/24h	FCC	1×10^{-3}	160 [b]	440	71	47
		AC + 1000°C/24h + compressed 40% + 1000°C/1h + 77K CrR (80%)	FCC	1×10^{-3}	1500	1500	12	47
		AC + 1000°C/24h + compressed 40% + 1000°C/1h + 293K CrR (80%)	FCC	1×10^{-3}	1200	1200	14	47
		AC + 1200°C/48h + CR (87%) + 800°C/1h	FCC, GS = 4.4 μm	1×10^{-3}	362	651	51	48
		AC + 1200°C/48h + CR (87%) + 1000°C/1h	FCC, GS = 50 μm	1×10^{-3}	197	568	60	48
		AC + 1200°C/48h + CR (87%) + 1150°C/1h	FCC, GS = 155 μm	1×10^{-3}	171	530	57	48
		AC + 1000°C/24h + 1000°C HR (92%) + 900°C/1h	FCC, GS = 32 μm	1×10^{-3}	223	587	39	40
		AC + 1200°C/48h + RS (60%) + 900°C/1h	FCC, GS = 17μm	1×10^{-3}	265 ± 10	600 ± 40	48 [b]	49
	$Co_{0.19}Cr_{0.08}Fe_{1.54}Mn_{1.04}Ni$	AC + 900°C HR (50%) + 1200°C/2h/WQ	FCC, GS = 24μm	2.5×10^{-3}	95	375	58	50
		AC + 900°C HR (50%) + 1200°C/2h/WQ + CR (64%)	FCC	2.5×10^{-3}	760	760	17	50
		AC + 900°C HR (50%) + 1200°C/2h/WQ + CR (64%) + 900°C/10min	FCC, GS = 12μm	2.5×10^{-3}	240	645	59 [b]	50
	$Co_{1.4}CrFeMnNi$	AC + 1000°C/24h/SC + CR (80%) + 1100°C/1h	FCC, GS = 60-80μm	7.3×10^{-4}	134	414	73.5	39
		AC + 1000°C/24h/SC + CR (80%) + 625°C/1h	FCC, GS = 1-2μm	7.3×10^{-4}	586	715	32.8	39
	$CoCr_{1.3}FeMnNi_{0.7}$	AC + 1000°C/24h/SC + CR (80%) + 1100°C/1h	FCC, GS = 60-80μm	7.3×10^{-4}	162	462	51.6	39
		AC + 1000°C/24h/SC + CR (80%) + 675°C/1h	FCC+σ, GS = 1-2μm	7.3×10^{-4}	1153	1187	1.8	39
CoCrFeMnNiV	CoCrFeMnNiV	AC	FCC+σ	1×10^{-3}		90	0	36
		AC + 1000°C/24h	FCC+σ	1×10^{-3}		62	0	36
CoCrFeNiTi	$Co_{1.5}CrFeNi_{1.5}Ti_{0.5}$	MA + SPS	2FCC+BCC	2.5×10^{-5}	1308	1384	4.01	51
	$Co_{1.5}CrFeNi_{1.5}Ti_{0.5}Mo_{0.1}$	AC	FCC+Ni_3Ti+ $(Cr_{11}Fe_{13}Ni_4)Mo_3$	5×10^{-5}	610	770	3.3	52
		SEBM	FCC+SC+Ni_3Ti	5×10^{-5}	750	930	4 [b]	52
		SEBM + 1120°C/3h	FCC+SC	5×10^{-5}	900 [b]	1320	18 [b]	52
		SEBM + 1120°C/3h/WQ	FCC+SC	5×10^{-5}	770	1120	36 [b]	52
CoCrFeNbNi	$CoCrFeNb_{0.103}Ni$	AC	FCC+LAVES	1×10^{-3}	317	622	19.2	37
	$CoCrFeNb_{0.155}Ni$	AC	FCC+LAVES	1×10^{-3}	321	744	23.3	37
	$CoCrFeNb_{0.206}Ni$	AC	FCC+LAVES	1×10^{-3}	402	807	8.6	37

	$CoCrFeNb_{0.309}Ni$	AC	FCC+LAVES	1×10^{-3}	478	879	3.5	37
	$CoCrFeNb_{0.412}Ni$	AC	FCC+LAVES	1×10^{-3}	637	1004	1.3	37
CoCrFeMoNi	$CoCrFeMo_{0.1}Ni$	AC + 500°C/4h	FCC	1×10^{-3}	198.8	479.0	51.1	38
	$CoCrFeMo_{0.2}Ni$	AC + 500°C/4h	FCC	1×10^{-3}	254.7	589.6	55.1	38
	$CoCrFeMo_{0.3}Ni$	AC + 500°C/4h	FCC+σ	1×10^{-3}	305.3	709.7	49.3	38
		AC + CR (60%) + 850°C/1h	FCC+σ+μ	1×10^{-3}	815.5	1186.5	18.9	38
		AC + CR (60%) + 950°C/5 h	FCC+σ+μ	1×10^{-3}	646.7	1042.0	32.5	38
		AC + CR (60%) + 950°C/5h + 700°C/5h	FCC+σ+μ	1×10^{-3}	683.7	1066.6	30.4	38
	$Co_{1.75}Cr_{0.75}FeMo_{0.5}Ni$	AC + 1200°C/48h/SC + 1100°C HR & CR (70%)	FCC	1×10^{-3}	350	720	21.9	53
		AC + 1200°C/48h/SC + 1100°C HR & CR (70%) + 800°C/1h/AC	FCC+μ	1×10^{-3}	1311	1410	12.1	53
		AC + 1200°C/48h/SC + 1100°C HR & CR (70%) + 850°C/5min/WQ	FCC+μ	1×10^{-3}	1212	1360	14.9	53
		AC + 1200°C/48h/SC + 1100°C HR & CR (70%) + 900°C/5min/WQ	FCC+μ	1×10^{-3}	1028	1249	18.3	53
		AC + 1200°C/48h/SC + 1100°C HR & CR (70%) + 1000°C/5min/WQ	FCC+μ	1×10^{-3}	879	1194	25.4	53
		AC + 1200°C/48h/SC + 1100°C HR & CR (70%) + 1000°C/1h/AC	FCC+μ	1×10^{-3}	799	1127	28.2	53
		AC + 1200°C/48h/SC + 1100°C HR & CR (70%) + 1150°C/1h/AC	FCC+μ	1×10^{-3}	350	918	62.4	53
	$Co_{2.125}Cr_{0.625}FeMo_{0.25}Ni$	AC + 1200°C/48h/SC + 1100°C HR & CR (70%)	FCC	1×10^{-3}	220 [b]	540 [b]	60 [b]	53
		AC + 1200°C/48h/SC + 1100°C HR & CR (70%) + 700°C/1h/AC	FCC	1×10^{-3}	800 [b]	1050 [b]	45 [b]	53
		AC + 1200°C/48h/SC + 1100°C HR & CR (70%) + 800°C/1h/AC	FCC	1×10^{-3}	570 [b]	950 [b]	55 [b]	53
		AC + 1200°C/48h/SC + 1100°C HR & CR (70%) + 1000°C/1h/AC	FCC	1×10^{-3}	370 [b]	810 [b]	72 [b]	53
CoCrFeNiV	CoCrFeNiV	AC	FCC+σ	1×10^{-3}		311	0	36
		AC + 1000°C/24h	FCC+σ	1×10^{-3}		330	0	36
CoCuFeNiSn	$CoCuFeNiSn_x$ ($0<x\leq0.2$)	AC	FCC ($0<x<0.04$) FCC+$Cu_{81}Sn_{22}$ ($0.04\leq x\leq0.2$)	2×10^{-4}	250-350 [b]	263(x=0.2)-633(x=0.07	1.2(x=0.2)-19.8(x=0.0	54

						)	5)	
CoCuFeMnNiSn	$CoCuFeMnNiSn_x$ (0<x≤0.2)	AC (DS)	FCC (0<x<0.05)<br>FCC+$Cu_{5.6}Sn$ (0.05≤x≤0.2)	$1×10^{-4}$	200-250 [b]	360(x=0.2)-476.9 (x=0.03) [b]	1.7(x=0.2)-16.9(x=0.03)	55
HfNbTiZr	HfNbTiZr	AC + 1300°C/6h/SC	BCC	$1×10^{-3}$	879	969	14.9	56
HfTaTiZr	HfTaTiZr	AC	BCC	$1×10^{-3}$	1356±86	1452±88	4±1.7	57
	$HfTa_{0.6}TiZr$	AC	BCC	$1×10^{-3}$	750±80	1110±85	22.1±1.6	57
	$HfTa_{0.5}TiZr$	AC	BCC+HCP	$1×10^{-3}$	687±23	1119±39	29.9±2.7	57
	$HfTa_{0.4}TiZr$	AC	BCC+HCP	$1×10^{-3}$	345±26	1126±55	30.6±1.7	57
HfNbTaTiZr	HfNbTaTiZr	AC	BCC	$5×10^{-3}$	790-805	857-888	5.8-9.4	58
		AC + HIP@1200°C/207MPa/2h + 1200°C/24h + CR (86.4%)	BCC	$1×10^{-3}$	1202	1295	4.7	59
		AC + HIP@1200°C/207MPa/2h + 1200°C/24h + CR (86.4%) + 800°C/2h/SC	BCC	$1×10^{-3}$	1303	1334	1.9	59
		AC + HIP@1200°C/207MPa/2h + 1200°C/24h + CR (86.4%) + 1000°C/2h/SC	BCC	$1×10^{-3}$	1145	1262	9.7	59
	$Hf_{0.5}Nb_{0.5}Ta_{0.5}Ti_{1.5}Zr$	AC	BCC	$1×10^{-3}$	903	990	18.8	60
	$HfNb_{0.2}Ta_{0.2}Ti_{1.3}Zr$	AC + CR (60%) + 900°C/30min	BCC+HCP	$1×10^{-4}$	540	995	23	61

a. The following acronyms are used: AC (as-cast); CR (cold-rolled); HR (hot-rolled); CrR (cryo-rolled); CF (cold forged); HF (hot forged); UF (upset forged); HD (hot drawing); RS (rotary swaged); SC (slow cooled); WQ (water quench); SLM (selective laser melting); HIP (hot isostatic pressing); DS (Directional solidification); MA (mechanically alloyed); SPS (spark plasma sintered); SEBM (selective electron beam melting); FSP (friction stir processing); EMS (electromagnetic stirring); GB (grain boundary); GS (grain size).

b. These data were measured from the tensile curves presented in the literature.

Supplementary References:

1 Kuznetsov, A. V. *et al.* Tensile properties of an AlCrCuNiFeCo high-entropy alloy in as-cast and wrought conditions. *Mater Sci Eng A* **533**, 107-118 (2012).

2 Wang, F., Zhang, Y. & Chen, G. Tensile and compressive mechanical behavior of a $CoCrCuFeNiAl_{0.5}$ high entropy alloy. *Int J Mod Phys B* **23**, 1254-1259 (2009).

3 Sheng, H. F. & Peng, L. M. Microstructure and tensile properties of $Al_{0.5}CoCrCuFeNi$ high-entropy alloy. *Appl Mech Mater* **456**, 494-497 (2014).

4 Tsai, C.-W., Tsai, M.-H., Yeh, J.-W. & Yang, C.-C. Effect of temperature on mechanical properties of $Al_{0.5}CoCrCuFeNi$ wrought alloy. *J Alloy Compd* **490**, 160-165 (2010).

5 Tsai, C. W. *et al.* Microstructure and tensile properties of $Al_{0.5}CoCrCuFeNi$ alloys produced by simple rolling and annealing. *Materials Science and Technology* **31**, 1178-1183 (2015).

6 Hemphill, M. A. *et al.* Fatigue behavior of $Al_{0.5}CoCrCuFeNi$ high entropy alloys. *Acta Mater* **60**, 5723-5734 (2012).

7 Du, Y. *et al.* Effect of electromagnetic stirring on microstructure and properties of $Al_{0.5}CoCrCuFeNi$ alloy. *Procedia Engineering* **27**, 1129-1134 (2012).

8 Wang, Z. G. *et al.* Effect of coherent $L1_2$ nanoprecipitates on the tensile behavior of a fcc-based high-entropy alloy. *Mater Sci Eng A* **696**, 503-510 (2017).

9 Daoud, H. M. *et al.* Microstructure and tensile behavior of $Al_8Co_{17}Cr_{17}Cu_8Fe_{17}Ni_{33}$ (at.%) high-entropy alloy. *JOM* **65**, 1805-1814 (2013).

10 Komarasamy, M. *et al.* Effect of Microstructure on the deformation mechanism of friction stir-processed $Al_{0.1}CoCrFeNi$ high entropy alloy. *Materials Research Letters* **3**, 30-34 (2014).

11 Kumar, N. *et al.* Friction stir processing of a high entropy alloy $Al_{0.1}CoCrFeNi$. *JOM* **67**, 1007-1013 (2015).

12 Hou, J., Zhang, M., Yang, H. & Qiao, J. Deformation behavior of $Al_{0.25}CoCrFeNi$ high-entropy alloy after recrystallization. *Metals* **7**, 111 (2017).

13 Shun, T.-T. & Du, Y.-C. Microstructure and tensile behaviors of FCC $Al_{0.3}CoCrFeNi$ high entropy alloy. *J Alloy Compd* **479**, 157-160 (2009).

14 Ma, S. G. *et al.* Superior high tensile elongation of a single-crystal $CoCrFeNiAl_{0.3}$ high-entropy alloy by Bridgman solidification. *Intermetallics* **54**, 104-109 (2014).

15 Joseph, J., Stanford, N., Hodgson, P. & Fabijanic, D. M. Tension/compression asymmetry in additive manufactured face centered cubic high entropy alloy. *Scripta Mater* **129**, 30-34 (2017).

16 Gwalani, B. *et al.* Optimizing the coupled effects of Hall-Petch and precipitation strengthening in a $Al_{0.3}CoCrFeNi$ high entropy alloy. *Mater Design* **121**, 254-260 (2017).

17 Rao, J. C. *et al.* Secondary phases in $Al_XCoCrFeNi$ high-entropy alloys: An in-situ TEM heating study and thermodynamic appraisal. *Acta Mater* **131**, 206-220 (2017).

18 Li, D. *et al.* High-entropy $Al_{0.3}CoCrFeNi$ alloy fibers with high tensile strength and ductility at ambient and cryogenic temperatures. *Acta Mater* **123**, 285-294 (2017).

19 Niu, S. *et al.* Strengthening of nanoprecipitations in an annealed $Al_{0.5}CoCrFeNi$ high entropy alloy. *Mater Sci Eng A* **671**, 82-86 (2016).

20 Lu, Y. *et al.* Directly cast bulk eutectic and near-eutectic high entropy alloys with balanced strength and ductility in a wide temperature range. *Acta Mater* **124**, 143-150 (2017).

21 Lu, Y. *et al.* A promising new class of high-temperature alloys: eutectic high-entropy alloys. *Scientific reports* **4**, 6200 (2014).

22 Wani, I. S. *et al.* Ultrafine-grained $AlCoCrFeNi_{2.1}$ eutectic high-entropy alloy. *Materials Research Letters* **4**, 174-179 (2016).

23 Wani, I. S. *et al.* Tailoring nanostructures and mechanical properties of $AlCoCrFeNi_{2.1}$ eutectic high entropy alloy using thermo-mechanical processing. *Mater Sci Eng A* **675**, 99-109 (2016).

24 Wang, Q. *et al.* A cuboidal B2 nanoprecipitation-enhanced body-centered-cubic alloy $Al_{0.7}CoCrFe_2Ni$ with prominent tensile properties. *Scripta Mater* **120**, 85-89 (2016).

25 Zhao, Y. L. *et al.* Heterogeneous precipitation behavior and stacking-fault-mediated deformation in a CoCrNi-based medium-entropy alloy. *Acta Mater* **138**, 72-82 (2017).

26 He, J. Y. *et al.* A precipitation-hardened high-entropy alloy with outstanding tensile properties. *Acta Mater* **102**, 187-196 (2016).

27 Zuo, T.-t., Ren, S.-b., Liaw, P. K. & Zhang, Y. Processing effects on the magnetic and mechanical properties of $FeCoNiAl_{0.2}Si_{0.2}$ high entropy alloy. *International Journal of Minerals, Metallurgy, and Materials* **20**, 549-555 (2013).

28 He, J. Y. *et al.* Effects of Al addition on structural evolution and tensile properties of the FeCoNiCrMn high-entropy alloy system. *Acta Mater* **62**, 105-113 (2014).

29 Ng, C. *et al.* Phase stability and tensile properties of Co-free $Al_{0.5}CrCuFeNi_2$ high-entropy alloys. *J Alloy Compd* **584**, 530-537 (2014).

30 Ma, S. G., Chen, Z. D. & Zhang, Y. Evolution of microstructures and properties of the $Al_XCrCuFeNi_2$ high-entropy alloys. *Mater Sci Forum* **745-746**, 706-714 (2013).

31 Dong, Y. *et al.* A multi-component $AlCrFe_2Ni_2$ alloy with excellent mechanical properties. *Mater Lett* **169**, 62-64 (2016).

32 Shaysultanov, D. G. *et al.* Novel $Fe_{36}Mn_{21}Cr_{18}Ni_{15}Al_{10}$ high entropy alloy with bcc/B2 dual-phase structure. *J Alloy Compd* **705**, 756-763 (2017).

33 Wang, Z., Baker, I., Guo, W. & Poplawsky, J. D. The effect of carbon on the microstructures, mechanical properties, and deformation mechanisms of thermo-mechanically treated $Fe_{40.4}Ni_{11.3}Mn_{34.8}Al_{7.5}Cr_6$ high entropy alloys. *Acta Mater* **126**, 346-360 (2017).

34 Wang, Z. & Baker, I. Effects of annealing and thermo-mechanical treatment on the microstructures and mechanical properties of a carbon-doped FeNiMnAl multi-component alloy. *Mater Sci Eng A* **693**, 101-110 (2017).

35 Brif, Y., Thomas, M. & Todd, I. The use of high-entropy alloys in additive manufacturing. *Scripta Mater* **99**, 93-96 (2015).

36 Salishchev, G. A. *et al.* Effect of Mn and V on structure and mechanical properties of high-entropy alloys based on CoCrFeNi system. *J Alloy Compd* **591**, 11-21 (2014).

37 Liu, W. H. *et al.* Effects of Nb additions on the microstructure and mechanical property of CoCrFeNi high-entropy alloys. *Intermetallics* **60**, 1-8 (2015).

38 Liu, W. H. *et al.* Ductile $CoCrFeNiMo_X$ high entropy alloys strengthened by hard intermetallic phases. *Acta Materialia* **116**, 332-342 (2016).

39 Zaddach, A. J., Scattergood, R. O. & Koch, C. C. Tensile properties of low-stacking fault energy high-entropy alloys. *Mater Sci Eng A* **636**, 373-378 (2015).

40 Gali, A. & George, E. P. Tensile properties of high- and medium-entropy alloys. *Intermetallics* **39**, 74-78 (2013).

41 Wu, Z., Bei, H., Pharr, G. M. & George, E. P. Temperature dependence of the mechanical properties of equiatomic solid solution alloys with face-centered cubic crystal structures. *Acta Mater* **81**, 428-441 (2014).

42 Deng, Y. *et al.* Design of a twinning-induced plasticity high entropy alloy. *Acta Mater* **94**, 124-133 (2015).

43 Li, Z., Tasan, C. C., Pradeep, K. G. & Raabe, D. A TRIP-assisted dual-phase high-entropy alloy: Grain size and phase fraction effects on deformation behavior. *Acta Mater* **131**, 323-335 (2017).

44 Li, Z. *et al.* Metastable high-entropy dual-phase alloys overcome the strength-ductility trade-off. *Nature* **534**, 227-230 (2016).

45 Wu, Z. & Bei, H. Microstructures and mechanical properties of compositionally complex Co-free $FeNiMnCr_{18}$ FCC solid solution alloy. *Mater Sci Eng A* **640**, 217-224 (2015).

46 Gludovatz, B. *et al.* A fracture-resistant high-entropy alloy for cryogenic applications. *Science* **345**, 1153-1158 (2014).

47 Stepanov, N. *et al.* Effect of cryo-deformation on structure and properties of CoCrFeNiMn high-entropy alloy. *Intermetallics* **59**, 8-17 (2015).

48 Otto, F. *et al.* The influences of temperature and microstructure on the tensile properties of a CoCrFeMnNi high-entropy alloy. *Acta Mater* **61**, 5743-5755 (2013).

49 Laplanche, G. *et al.* Microstructure evolution and critical stress for twinning in the CrMnFeCoNi high-entropy alloy. *Acta Mater* **118**, 152-163 (2016).

50 Yao, M. J., Pradeep, K. G., Tasan, C. C. & Raabe, D. A novel, single phase, non-equiatomic FeMnNiCoCr high-entropy alloy with exceptional phase stability and tensile ductility. *Scripta Mater* **72-73**, 5-8 (2014).

51 Moravcik, I. *et al.* Microstructure and mechanical properties of $Ni_{1.5}Co_{1.5}CrFeTi_{0.5}$ high entropy alloy fabricated by mechanical alloying and spark plasma sintering. *Mater Design* **119**, 141-150 (2017).

52 Fujieda, T. *et al.* CoCrFeNiTi-based high-entropy alloy with superior tensile strength and corrosion resistance achieved by a combination of additive manufacturing using selective electron beam melting and solution treatment. *Mater Lett* **189**, 148-151 (2017).

53 Ming, K., Bi, X. & Wang, J. Precipitation strengthening of ductile $Cr_{15}Fe_{20}Co_{35}Ni_{20}Mo_{10}$ alloys. *Scripta Mater* **137**, 88-93 (2017).

54 Liu, L. *et al.* Microstructure and the properties of $FeCoCuNiSn_X$ high entropy alloys. *Mater Sci Eng A* **548**, 64-68 (2012).

55 Liu, L. *et al.* Microstructure and tensile properties of $FeMnNiCuCoSn_X$ high entropy alloys. *Mater Design* **44**, 223-227 (2013).
56 Wu, Y. D. *et al.* A refractory $Hf_{25}Nb_{25}Ti_{25}Zr_{25}$ high-entropy alloy with excellent structural stability and tensile properties. *Mater Lett* **130**, 277-280 (2014).
57 Huang, H. *et al.* Phase-Transformation Ductilization of Brittle High-Entropy Alloys via Metastability Engineering. *Adv Mater* (2017).
58 Dirras, G. *et al.* Elastic and plastic properties of as-cast equimolar TiHfZrTaNb high-entropy alloy. *Mater Sci Eng A* **654**, 30-38 (2016).
59 Senkov, O. N. & Semiatin, S. L. Microstructure and properties of a refractory high-entropy alloy after cold working. *J Alloy Compd* **649**, 1110-1123 (2015).
60 Sheikh, S. *et al.* Alloy design for intrinsically ductile refractory high-entropy alloys. *J Appl Phys* **120**, 164902 (2016).
61 Lilensten, L. *et al.* Design and tensile properties of a bcc Ti-rich high-entropy alloy with transformation-induced plasticity. *Materials Research Letters* **5**, 110-116 (2016).
